# Comparative analysis of tissue-specific transcriptomic responses to nitrogen stress in spinach *(Spinacia oleracea)*

**DOI:** 10.1371/journal.pone.0232011

**Published:** 2020-05-06

**Authors:** Vijay Joshi, Madhumita Joshi, Arianne Penalosa

**Affiliations:** 1 Texas A&M AgriLife Research and Extension Center, Uvalde, Texas, United States of America; 2 Department of Horticultural Sciences, Texas A&M University, College Station, Texas, United States of America; 3 College of Science, University of Texas, Arlington, Texas, United States of America; ICAR-Indian Institute of Agricultural Biotechnology, INDIA

## Abstract

Nitrogen (N) is critical to the growth and productivity of crops. To understand the molecular mechanisms influenced by N stress, we used RNA-Sequencing (RNA-Seq) to analyze differentially expressed genes (DEGs) in root and leaf tissues of spinach. N stress negatively influenced photosynthesis, biomass accumulation, amino acid profiles, and partitioning of N across tissues. RNA-seq analysis revealed that N stress caused most transcriptomic changes in roots, identifying 1,346 DEGs. High-affinity nitrate transporters (NRT2.1, NRT2.5) and glutamine amidotransferase (GAT1) genes were strongly induced in roots in response to N deplete and replete conditions, respectively. GO and KEGG analyses revealed that the functions associated with metabolic pathways and nutrient reservoir activity were enriched due to N stress. Whereas KEGG pathway enrichment analysis indicated the upregulation of DEGs associated with DNA replication, pyrimidine, and purine metabolism in the presence of high N in leaf tissue. A subset of transcription factors comprising bHLH, MYB, WRKY, and AP2/ERF family members was over-represented in both tissues in response to N perturbation. Interesting DEGs associated with N uptake, amino acid metabolism, hormonal pathway, carbon metabolism, along with transcription factors, were highlighted. The results provide valuable information about the underlying molecular processes in response to N stress in spinach and; could serve as a resource for functional analysis of candidate genes/pathways and enhancement of nitrogen use efficiency.

## Introduction

Nitrogen (N), being a constituent of amino acids, nucleic acids, cofactors, and secondary metabolites, is central to most biological activities in plants. Effects of N on plant morphology rely on its concentration, which is a determining factor in several metabolic processes, resource allocation, growth, and development of plants [1]. N moves along a complex branching and merging pathway which interacts at numerous sites with the carbon (C) flow, ion, and assimilates at the cell and whole plant level [2]. N is typically transported in the form of nitrate (NO_3_^−^), dissolved ammonia, and amino acids. Most of the NO_3_^−^ is reduced in the shoots, while ammonia incorporates into amino acids for long-distance translocation. Among various external factors that affect plant growth, N partitioning, in particular, defines the productivity by altering the ratios of roots to shoots [3, 4]. For example, in spinach (*Spinacia oleracea*), suboptimal N nutrition enhanced root growth at the expense of shoot biomass [5, 6].

Spinach is one of the popular nutritious green leafy vegetables grown in most parts of the world. Although understanding the mechanisms of N metabolism in spinach is of considerable significance, most of the previous studies have mainly focused on physiological and agronomical aspects. Most plants can consume only half of the applied N, losing it in the form of NO_3_^−^, which leads to groundwater contamination, health, and environmental hazards [7, 8]. In commercial spinach production, it is estimated that about 60% of N is lost through leaching [9], most likely due to its shallow root system [10] and short production cycle. Although spinach needs significant amounts of N to sustain rapid growth, it is relatively weak in NO_3_^−^ reduction [11–13]. One way to tackle this crisis without compromising productivity is to improve nitrogen use efficiency (NUE) [14].

N perturbation induces series of molecular changes associated with intrinsic processes such as chlorophyll synthesis [15], root architecture [16], and metabolism of sugars and sugar phosphates [17]. In particular, N starvation results in diminished levels of N containing metabolites such as amino acids glutamate (Glu) and glutamine (Gln) [18]. As plants take up NO_3_^−^ and ammonium (NH_4_^+^) from the soil and assimilate into amino acids in roots/shoots, improving the amino acid partitioning from source to sink would be an effective strategy to optimize N utilization [19]. To date, quite a few studies have been conducted to investigate genome-wide expression analysis in response to N stress in various plants such as *Arabidopsis* [20–22], rice [23–25], maize [26, 27], wheat [28, 29], and *Brassica* species [30]. Although this information should help in understanding general molecular processes, each species has its unique and non-overlapping mechanisms to withstand N stress. Besides few studies focusing on mechanisms of N uptake [31, 32] and genotypic variation [6, 33, 34], not much is known about the transcriptomic changes in the vegetative or non-vegetative tissues of spinach in response to N perturbations.

Here we performed RNA-sequencing (RNA-Seq) of leaf and root tissues of spinach combined with physiological and metabolic analyses using two N treatments: high N (200 ppm) and low N (50 ppm). A total of 1346 and 1136 differentially expressed genes (DEGs) were identified in response to N in root and leaf tissues, respectively. The changes in the expression of the identified genes and the impacted pathways in response to N would help in advancing our understanding of tissue-specific molecular and physiological processes in spinach. The novel genes/pathways identified in this study have provided new targets for genetic manipulation or introgression breeding to improve NUE in spinach.

## Materials and methods

### Plant material and growth conditions

The spinach plants were grown in an environmentally controlled growth chamber at the Texas A&M AgriLife Research and Extension Center, Uvalde, Texas. The seeds of spinach variety ‘Banjo’ were grown in plastic containers containing Turface ® based growing medium under 200 μmol·mPPPPP^-2^PPPPP·s PPPPP^-1^ PPPPPlight intensity PPPPP PPPPP (12 hours each light and dark period) at 23°C and 60–70% relative humidity. Two treatments, high N and low N, where N was added in the form of Ca(NO_3_)_2_, were used to maintain the concentration of 200 ppm for high N and 50 ppm for low N. CaCl_2_ was compensated to keep the same concentration of calcium in both treatments. The concentrations of remaining macro- and micro-elements were kept constant using N-free Hoagland’s nutrient solution (No. 2 Basal Salt Mixture, Caisson Labs, Smithfield, UT, USA) comprising 2.86 mg·LPPPPP^−1^PPPPP H_3_BO_3_, 554.90 mg·LPPPPP^−1^PPPPP CaCl_2_R, 0.045 mg·LPPPPP^−1^PPPPP CuCl_2_, 33.0 mg·LPPPPP^−1^PPPPP C_10_RRH_12_N_2_ NaFeO_8_·3H_2_O, 240.33 mg·LPPPPP^−1^PPPPP MgSO_4_, 1.81 mg·LPPPPP^−1^PPPPP MnCl_2_R·4H_2_O, 0.025 mg·LPPPPP^−1^PPPPP Na_2_MoO_4_·2H_2_O, 372.70 mg·LPPPPP^−1^PPPPP KCl, 136.025 mg·LPPPPP^−1^PPPPP KH_2_PO_4_, and 0.1 mg·LPPPPP^−1^PPPPP ZnCl_2_R. Samples collected from 6-week old plants were frozen in liquid nitrogen and stored at −80°C until subsequent analyses. Three independent plants were used for metabolic analysis, total RNA extractions, physiological and biochemical analysis.

### Determination of physiological traits, N, and free amino acids

Leaf photosynthetic rate (Pn), transpiration rate (Tr), stomatal conductance (Cs), and intercellular CO_2_ concentration were measured with LICOR-6400-XT Photosynthetic system (Lincoln, USA) from the fully expanded leaves of 6-week old plants. The chlorophyll content was measured using a portable chlorophyll meter (SPAD-502, Konica Minolta, Tokyo, Japan). The plant samples were analyzed for total N (TKN), NO_3_^−^, NH_4_^+^ Rusing Easy Chem Plus analyzer (Chinchilla Scientific, Oak Brook, IL). The amino acid analysis was performed using WatersPPPPP ^R^PPPPP Acquity H-class UPLC system coupled to WaterPPPPP^R^PPPPP’s Xevo TQs MS-MS mass spectrometer with electrospray ionization (ESI) probe following pre-established protocol [35]. Data integration and quantitation were performed using the Water’s TargetLynx^™^ software. Statistical analysis was performed using JMP Pro 14 (SAS Institute, Cary, NC, USA).

### RNA extraction, cDNA library preparation, and sequencing

#### Sample collection and preparation

The flash-frozen plant samples in liquid nitrogen were ground to a fine powder using a paint shaker (Harbil, Wheeling, IL, USA) and 3-mm-diameter steel balls (Abbott Ball, West Hartford, CT, USA). Total RNA was extracted using an RNeasy® Plant Mini Kit (QIAGEN Sciences, Germantown, MD, USA) as per the manufacturer’s protocol and treated with DNase1 (QIAGEN Sciences, Germantown, MD, USA). The purity of the RNA was analyzed using the NanoPhotometer® spectrophotometer (IMPLEN, CA, USA). RNA integrity and quantitation were assessed using the RNA Nano 6000 Assay Kit of the Bioanalyzer 2100 system (Agilent Technologies, CA, USA).

#### Library preparation for transcriptome sequencing

One μg total RNA per sample was used for the RNA library preparations. Sequencing libraries were generated using NEBNext® Ultra^™^ RNA Library Prep Kit for Illumina® (NEB, USA) following the manufacturer’s recommendations, and index codes were added to attribute sequences to each sample. Library concentration was first quantified using a Qubit 2.0 fluorometer (Life Technologies), diluted to 1 ng/μl before checking the insert size on an Agilent Bioanalyzer 2100 system and quantified to greater accuracy by quantitative PCR (Q-PCR) (library activity >2 nM).

#### Data processing and analysis

*Clustering*, *sequencing*, *and quality control*. The clustering of the index-coded samples was performed on a cBot Cluster Generation System using PE Cluster Kit cBot-HS (Illumina) according to the manufacturer’s instructions. After cluster generation, the libraries were sequenced on an Illumina Hiseq platform, and 150 bp paired-end reads were generated. Raw reads of fastq format were processed to obtain clean reads by removing the adapter, reads containing ploy N, and low-quality reads from raw data. At the same time, Q20, Q30, and GC content, the clean data were calculated.

*Reads mapping to the reference genome*. Reference genome and gene model annotation files were downloaded from SpinachBase (http://spinachbase.org/). Index of the reference genome was built using Bowtie v2.2.3, and paired-end clean reads were aligned to the reference genome using TopHat v2.0.12.

Gene expression quantification and DEG analysis:

HTSeq v0.6.1 was used to count the reads mapped to each gene. FPKM [36] of each gene was calculated based on the length of the gene and reads count mapped to this gene. Differential expression analysis of high N and low N conditions (three biological replicates per tissue per treatment) was performed using the DESeq R package (1.18.0) [37]. Genes with P-value < 0.05 found by DESeq were assigned as differentially expressed.

*GO and KEGG enrichment analysis*. Gene Ontology (GO) [38] enrichment analysis of differentially expressed genes was implemented by the GOseq R package, in which gene length bias was corrected. GO terms with P-value less than 0.05 were considered significantly enriched by DEGs. KOBAS software was used to test the statistical enrichment of differential expression genes in the Kyoto encyclopedia of genes and genome pathways database (KEGG; http://www.genome.jp/kegg/) [39].

#### Validation by quantitative real-time PCR

To validate the RNA-seq data, quantitative real-time PCR (RT-qPCR) was conducted to examine the expression pattern of twenty DEGs. Primer Premier 3.0 software was used to design gene-specific primers on the basis of the selected unigene sequences (S1 Table). Total RNA was extracted with the Quick-RNA^™^ Miniprep Kit (Zymo Research Corporation, Irvine, CA) treated with DNase1 (Zymo Research Corporation, Irvine, CA), and subjected to reverse transcription using iScript RT Supermix (Bio-Rad Laboratories, Inc, Hercules, USA). The quality and quantity of the RNA were analyzed by a Denovix DS-11+ spectrophotometer (Wilmington, Delaware, USA). Gene expression analysis via reverse transcription-qPCR was performed using the BioRad CFX96 qPCR instrument using SsoAdv Univer SYBR GRN Master Kit (Bio-Rad Laboratories, Inc, Hercules, USA). The expression levels of selected DEGs were normalized by comparing with an internal reference gene, 18SrRNA [40]. The relative expression levels (Cq values) for each gene were normalized to that of reference genes by taking an average of three biological replicates. The relative expression levels were calculated using the 2^−ΔΔCt^ method [41]. All RT-qPCR were repeated in three biological and three technical replications.

## Results and discussions

### Validation of N stress for expression profiling in spinach

**Fig 1:**
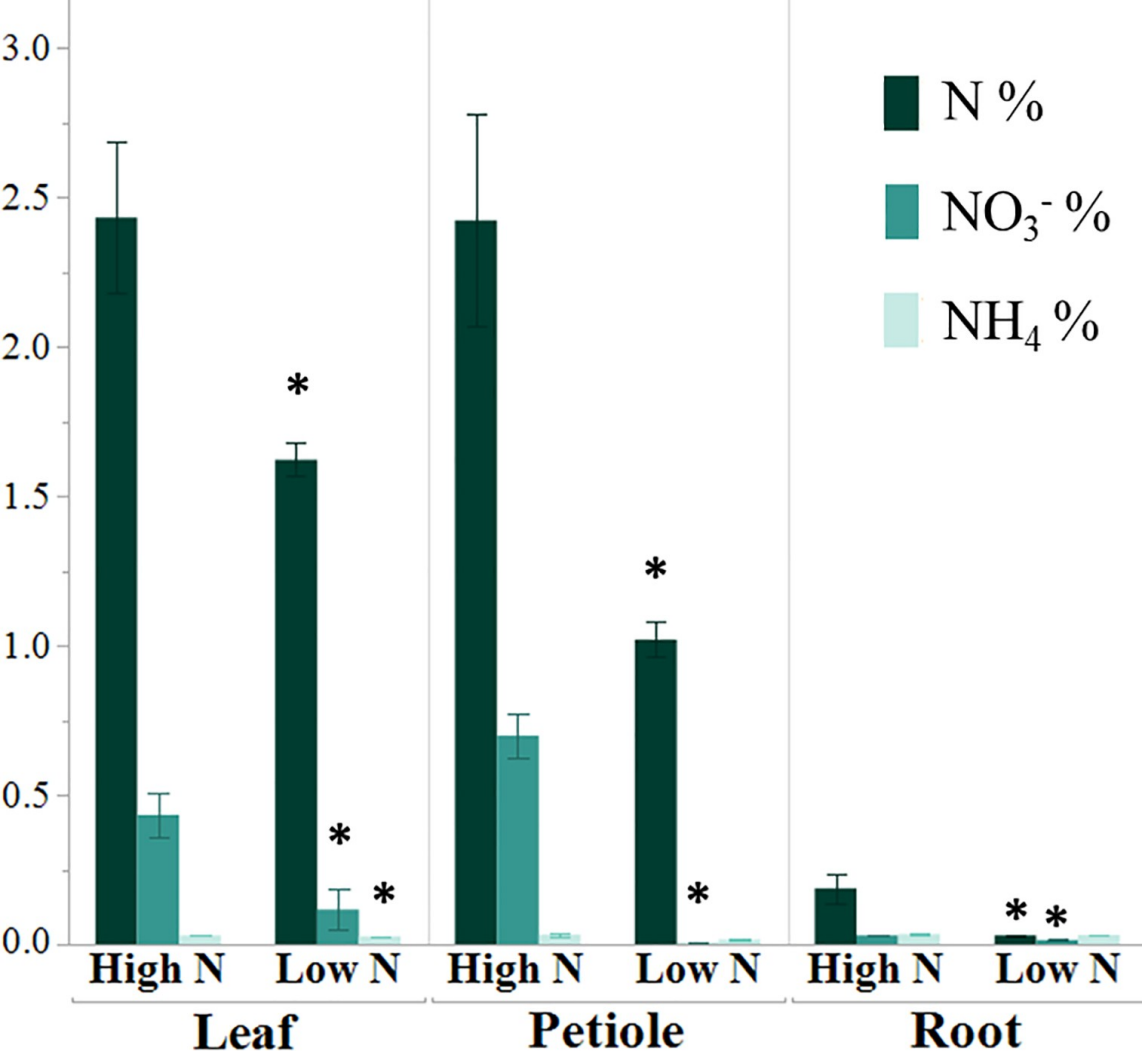
Percent changes in N content in spinach tissues due to N stress. (n = 3, Mean ± SE) * significant at P<0.05.

**Fig 2:**
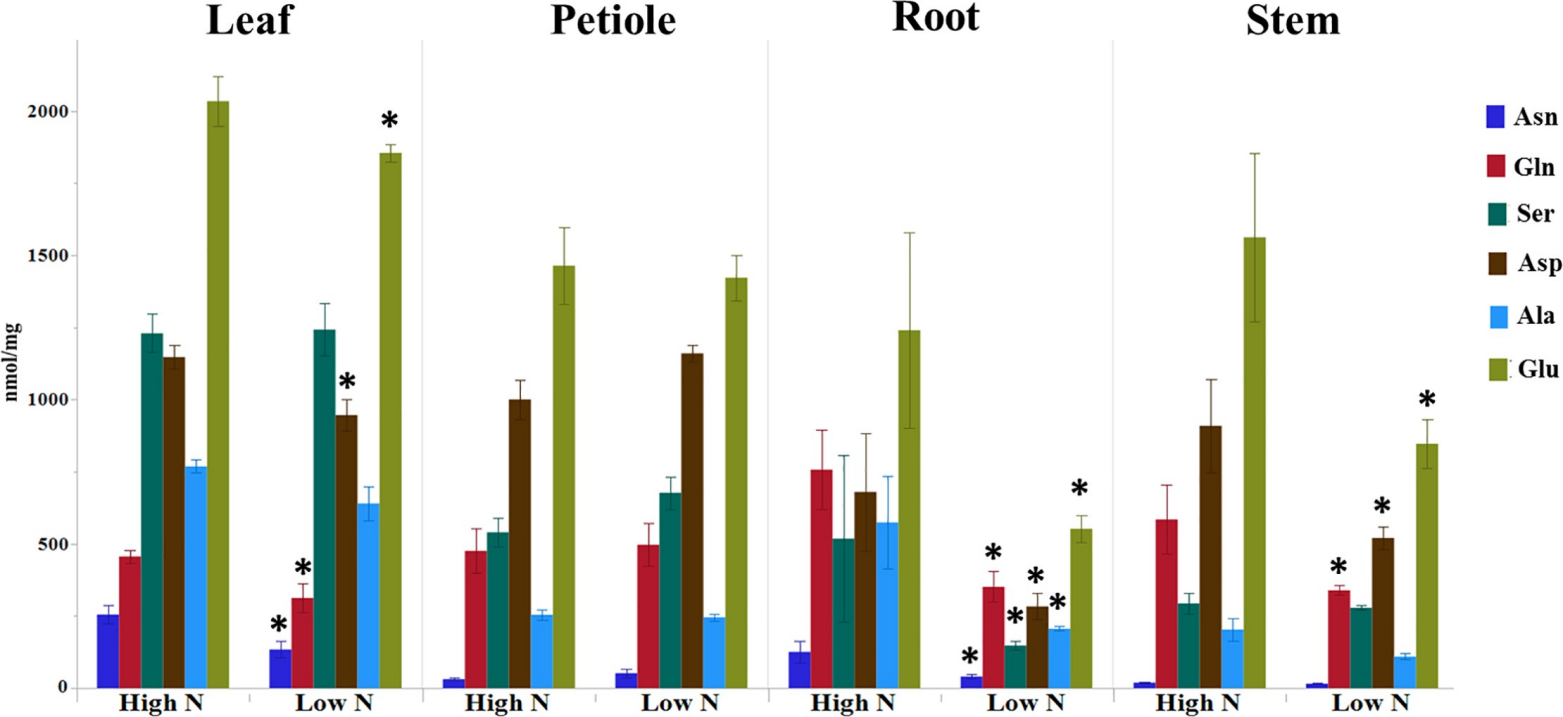
Changes in free amino acids in spinach due to N stress. Asparagine (Asn), Aspartate (Asp), Glutamine (Gln), Glutamate (Glu), Serine (Ser), and Alanine (Ala) (n = 3, Mean ± SE) * significant at P<0.05.

N availability affects its acquisition and metabolism in plants. Hence a detailed phenotypic characterization preceded sampling of material for RNA-Seq analysis. A generalized response to N limitation was confirmed by evaluating responses of gas exchange parameters in the leaves of 6-week old spinach plants. N stress significantly reduced the net photosynthetic rate, stomatal conductance, and transpiration rate, implying compromised photosynthetic performance (Table 1). Further, elemental N and NO_3_^−^_was_ significantly reduced in leaf, petiole, and root tissues validating treatment effect (Fig 1). NH_4_^+^ content was also lower in leaf tissues due to N stress. As most N in plants is converted to organic form as free amino acids, we compared the amounts of six highly abundant amino acids; Asparagine (Asn), Aspartate (Asp), Gln, Glu, Serine (Ser) and Alanine (Ala) in four tissue types of spinach. The contents of all the amino acids showed a significant reduction in the roots, while foremost N carrying amino acids (Asp, Asn, Glu, Gln) were significantly decreased in the leaf tissue (Fig 2). Unlike total N and content of NO_3_^−^, no significant changes were observed in amino acids in petiole due to N stress.

**Table 1:** Responses of gas exchange parameters to N stress in spinach.

Treatment	*P*_*n*_ (μmol CO_2_ m^-2^s^-1^)	*G*_*s*_ (mol_2_ H_2_O m^-2^ s^-1^)	*C*_*i*_ (μmol of CO_2_ / mol)	*E* (mmol H_2_O m^-2^ s^-1^)	SPAD
high N	9.79 ± 0.42	0.58 ± 0.04	347.1 ± 2.12	5.56 ± 0.27	43.7 ± 2.19
low N	6.01 ± 0.46	0.26 ± 0.05	341.2 ± 5.13	3.79 ± 0.5	35.8 ± 1.15
p-Value	0.00*	0.01*	0.34	0.02*	0.03*

*P*_*n*_*—*net photosynthetic rate, *E*- transpiration rate, *G*_*s*_*—*stomatal conductance, *C*_*i*_*—*intercellular CO_2_ concentration, SPAD (Soil Plant Analysis Development) units * significant at *P*<0.05.

### Transcriptome sequencing and assembly of sequencing data

The transcriptomic changes induced by N stress in a spinach leaf and root tissues were analyzed by RNA-Seq. Total twelve independent libraries; six from leaves (HNL1, HNL2, HNL3 for high N and LNL1, LNL2, LNL3 for low N) and six from roots (HNR1, HNR2, HNR3 for high N and LNR1, LNR2, LNR3 for low N) were sequenced using the Illumina HiSeq platform. On average, 45.53 to 43.47 million raw reads were generated for leaf and root tissues in both N treatments (Table 2). Across all reads, the Q20 and Q30 percentage was more than 97 and 93%, respectively (sequencing error rate was less than 0.03%), and GC content for the libraries was more than 43%. Additional significant characteristics of these libraries are summarized in S2 Table. Among all the libraries, the ratio of total mapped reads and multiple mapped reads in both tissue types was above 85% and 2.5%, respectively. Average 85% reads were uniquely mapped to the reference genome in both tissue types under both N treatments. The data generated from all libraries provided a foundation for quality analyses. The RNA-Seq dataset is accessible through GEO Series accession number GSE145943 (https://www.ncbi.nlm.nih.gov/geo/).

**Table 2:** Summary of sequencing data quality of spinach samples in both tissue types and N treatments.

Treatment	Tissue	Sample name	Raw reads	Clean reads	Error rate (%)	Q20 (%)	Q30 (%)	GC content (%)
high N	Leaf	HNL1	41597938	40201618	0.03	97.76	93.65	44.15
		HNL2	47471760	45594984	0.03	97.72	93.64	44.48
		HNL3	47246618	45862032	0.03	97.53	93.07	44.20
	Root	HNR1	43542944	42695366	0.03	97.66	93.34	43.31
		HNR2	42288790	41132378	0.03	97.52	93.08	43.21
		HNR3	42159430	39940286	0.03	97.60	93.30	43.22
low N	Leaf	LNL1	46979108	42951112	0.03	97.75	93.62	44.14
		LNL2	43156630	41716986	0.03	97.85	93.81	44.62
		LNL3	46758928	45391850	0.03	97.52	93.10	44.15
	Root	LNR1	45260090	44242952	0.03	97.71	93.49	43.29
		LNR2	46795700	45438476	0.03	97.72	93.49	43.57
		LNR3	40777766	39803014	0.03	97.90	93.95	43.33

The correlations among biological replicates were assessed using the Pearson correlation coefficient (S1 Fig) to validate the reliability of RNA-seq data. The libraries for the same treatment (i.e., biological replicates) were highly correlated. The weak correlation between tissue types within treatments suggests a significant impact of N stress on gene expression profiles. Distinct gene expression levels under different experiment conditions also suggest that root tissue is more sensitive to N perturbation than leaf tissue (S2 Fig).

### Analysis of differentially expressed genes (DEGs)

**Fig 3:**
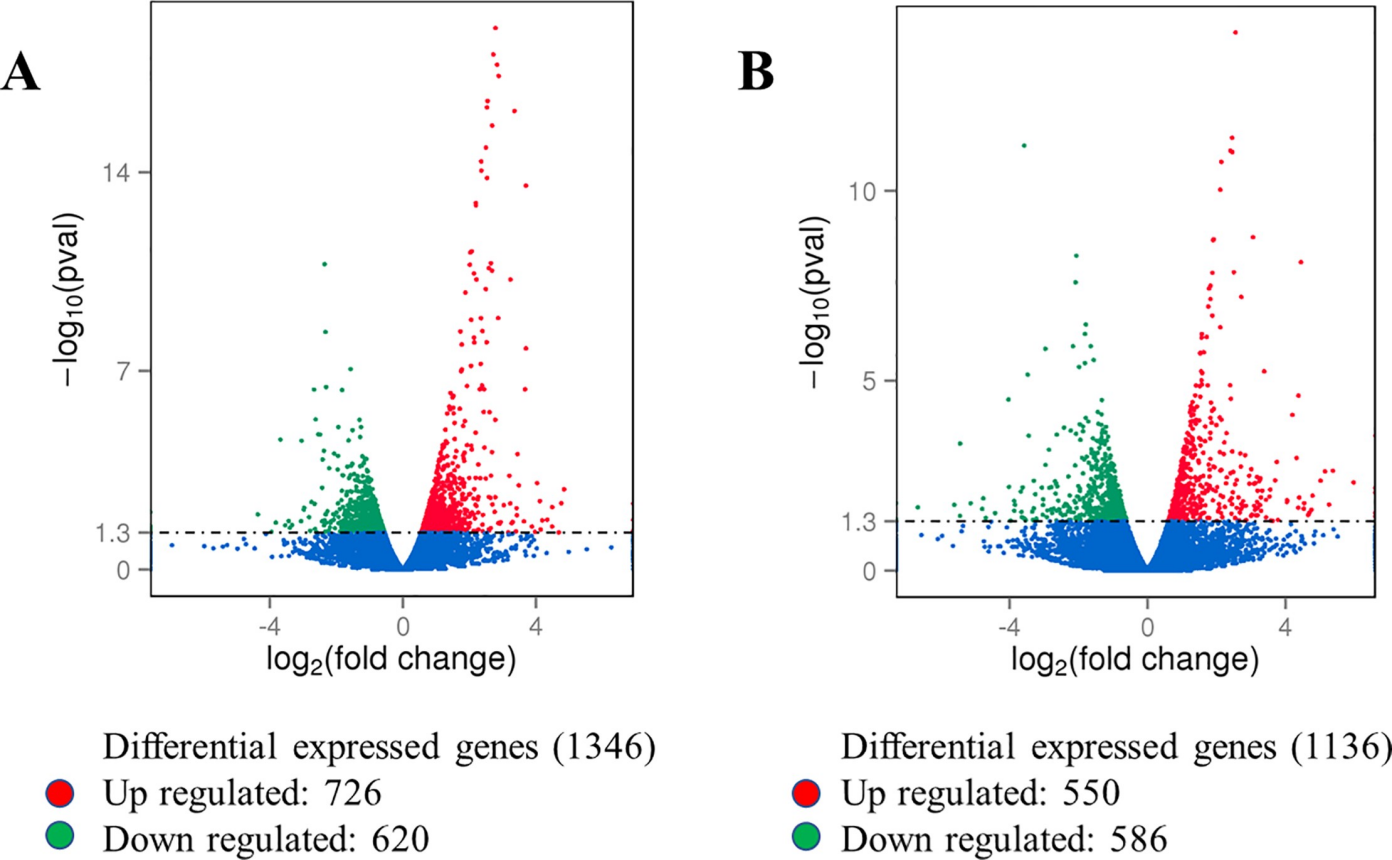
The volcano maps showing the number of differentially expressed genes (DEGs) in the root (A) and leaf (B) tissues in high N. Red dots represent up-regulated genes, and green dots represent down-regulated genes (p < 0.05).

**Fig 4:**
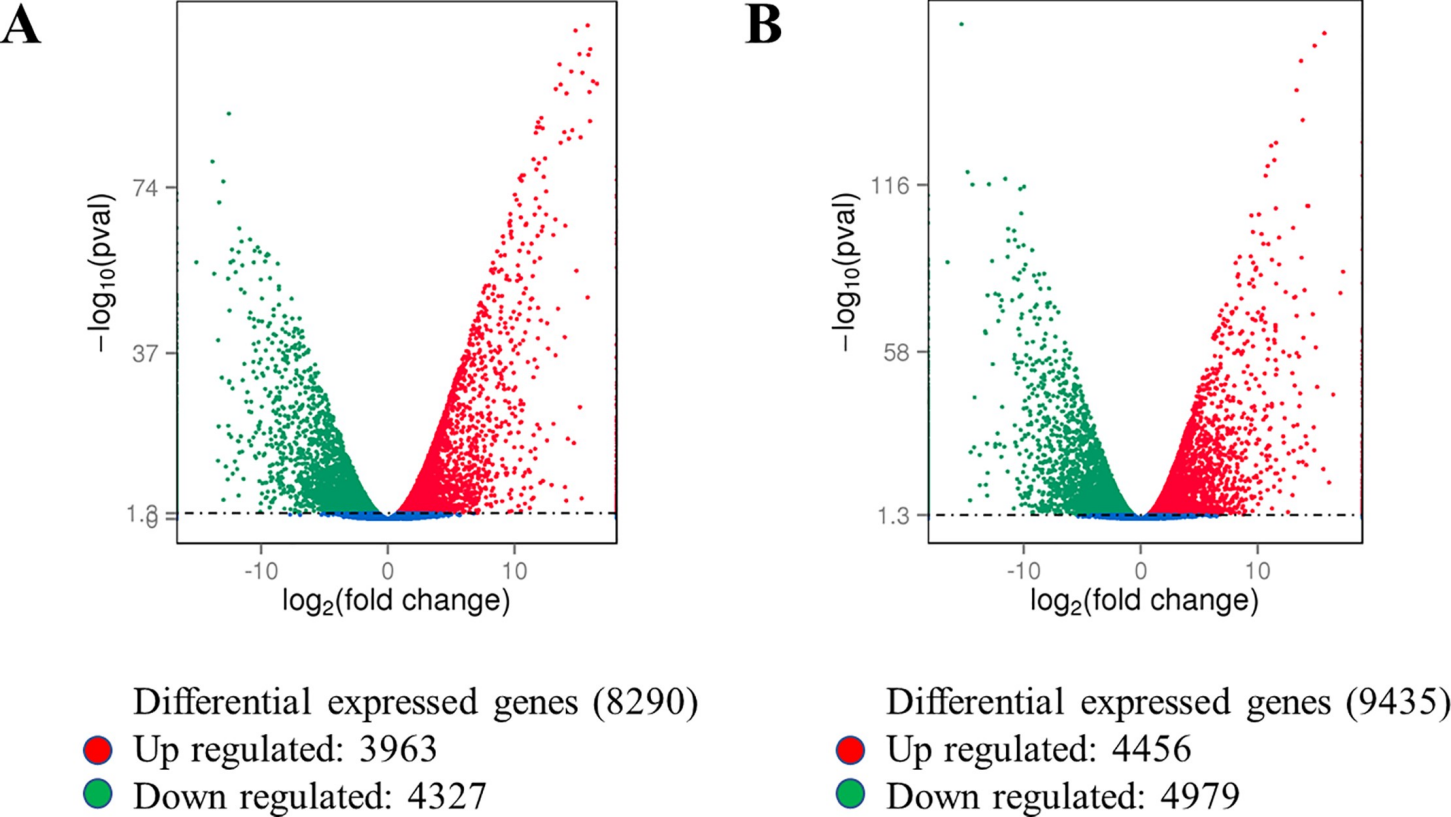
The volcano maps showing the number of differentially expressed genes (DEGs) in the leaf of spinach plants grown under low N (A) and high N (B) treatments. Red dots represent up-regulated genes, and green dots represent down-regulated genes (p < 0.05).

**Fig 5:**
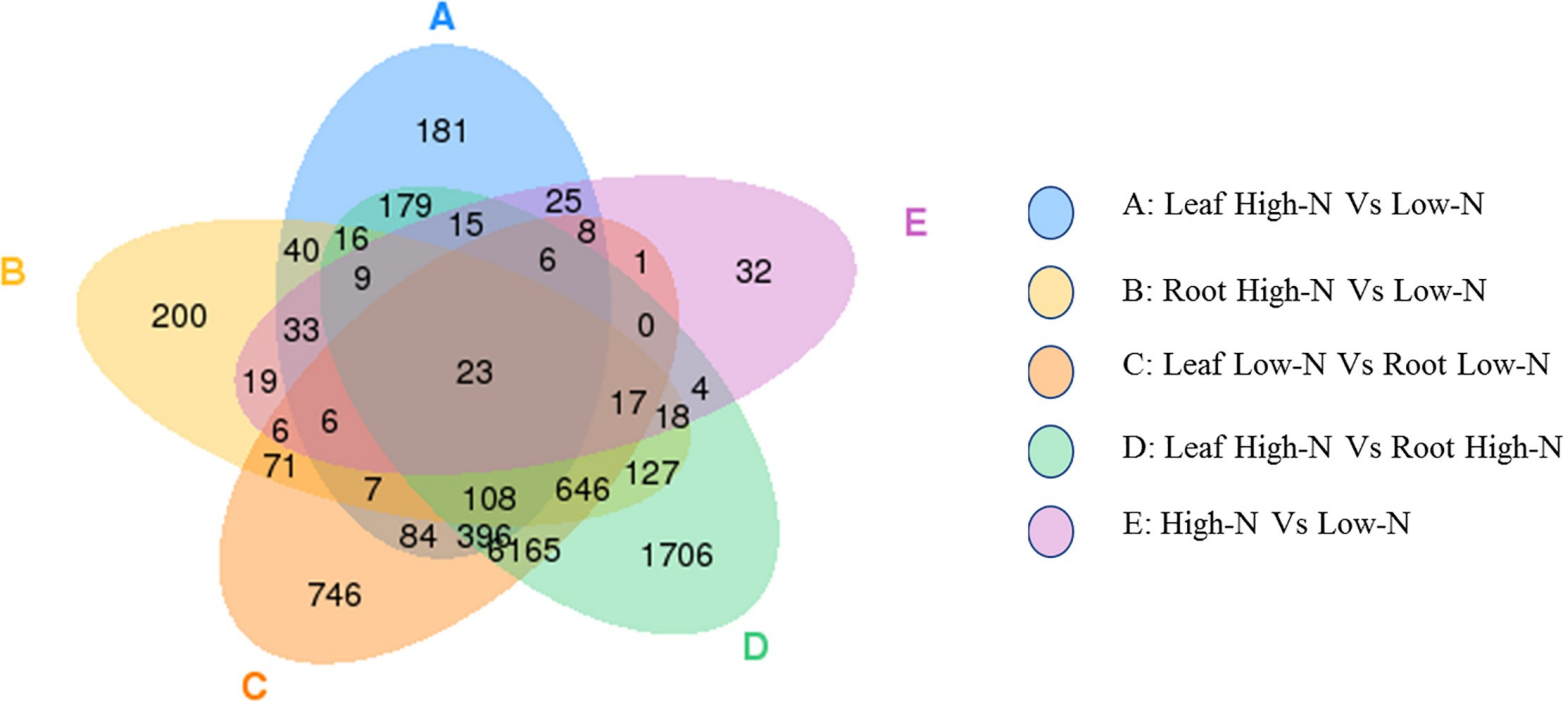
The Venn diagram presenting the number of differentially expressed genes (DEGs) within the treatment. The sum of the number in the circle presents the total number of DEGs, and the overlap presents the genes in common.

The cluster analysis confirmed that a large number of genes were differentially expressed in leaf and root tissues in both treatments (low N and high N) (S3 Fig). The DEG analysis of roots revealed that 1346 transcripts were significantly (p < 0.05) altered when plants were grown in high N. These included 726 upregulated and 620 downregulated transcripts (Fig 3, S3 Table, S4 Table). A total of 1136 genes were differentially expressed in leaf tissue, of which 550 genes were upregulated (Fig 3, S5 Table), while 586 were downregulated in high N (Fig 3, S6 Table). Within treatments, the number of DEGs was higher in high N treatment while within tissue types, it was more abundant in root tissue in both N treatments (Fig 4). Between treatments, the DEG analysis revealed that expression of 222 genes was significantly (p < 0.05) affected by N stress, of which 88 genes were upregulated and 134 genes downregulated in the presence of high N availability (S4 Fig). Together, all the DEGs in root and leaf tissues under both treatments are presented as a single Venn diagram (Fig 5). Total 200 and 181 DEGs were uniquely expressed in root and leaf tissue, respectively.

### Functional annotation and GO enrichment analysis of DEGs

**Fig 6:**
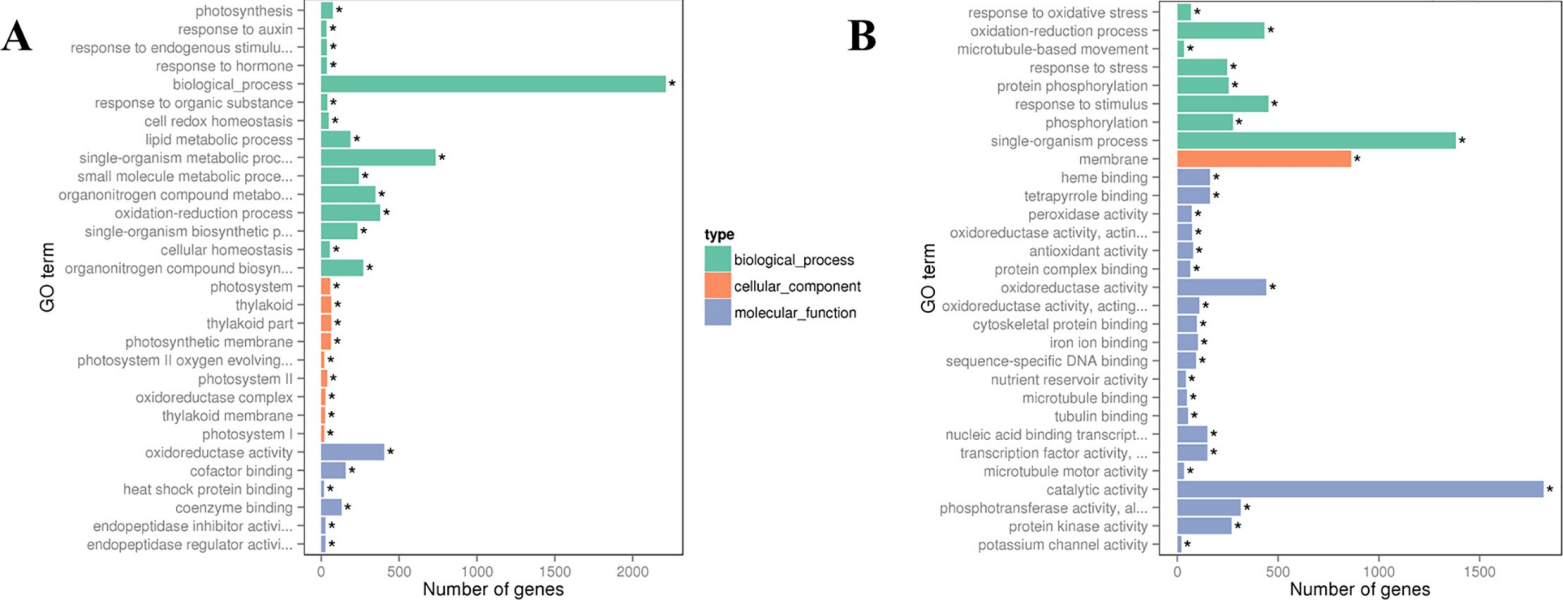
Gene ontology (GO) enrichment bar chart of DEGs showing most enriched G) terms of (A) upregulated and (B) downregulated DEGs in leaf under Low-N. The y-axis is the enriched GO term; the x-axis is the number of DEGs enriched in the listed term. Colors represent different GO types: biological process, cellular component, and molecular function. * significantly enriched term (p-value < 0.05).

**Fig 7:**
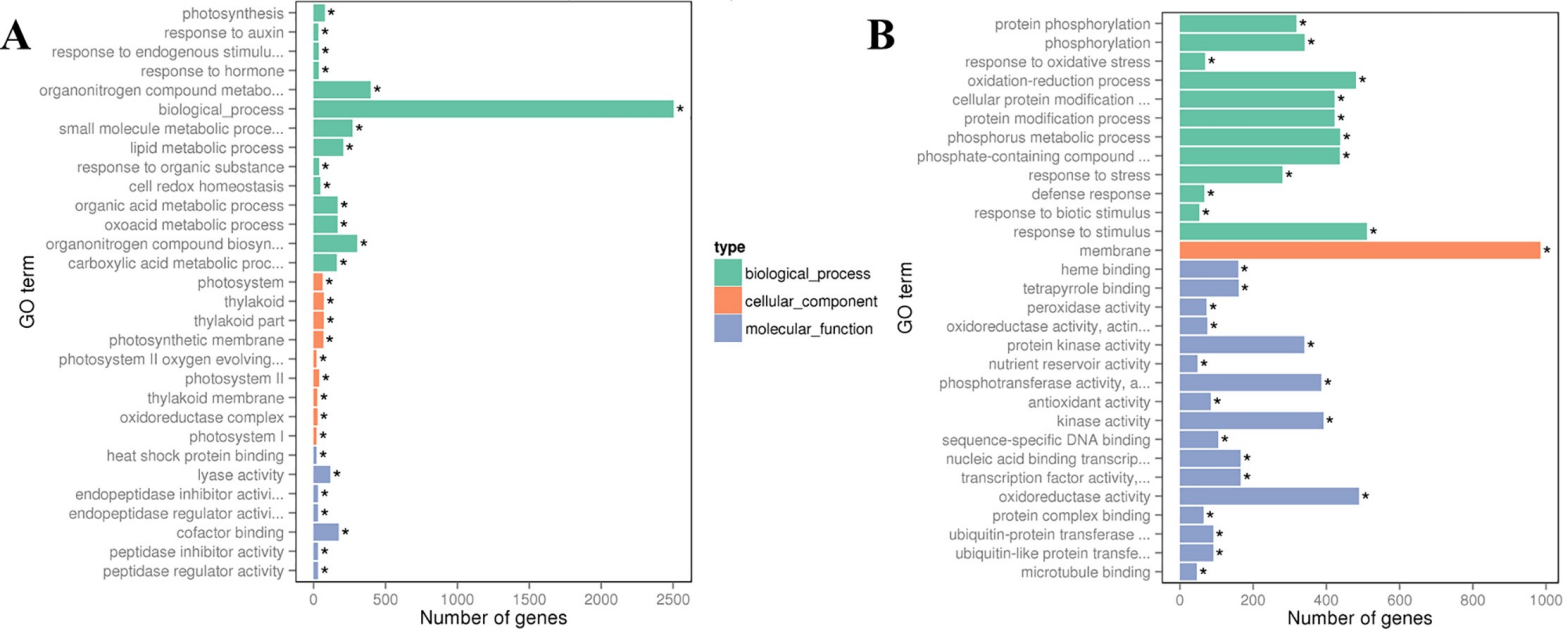
Gene ontology (GO) enrichment bar chart of DEGs showing most enriched G) terms of (A) upregulated and (B) downregulated DEGs in leaf tissues under High-N. The y-axis is the enriched GO term, and the x-axis is the number of DEGs enriched in the listed term. Colors represent different GO types: biological process, cellular component, and molecular function. * significantly enriched term (p-value < 0.05).

Among the DEGs that were significantly upregulated in leaf under Low N, functional annotation showed that 15 biological processes, 9 cell component metabolic pathways, and 6 molecular functions were significantly over-represented (p-value < 0.05) (Fig 6). Similarly, DEGs showing down-regulation in leaf affected 21 molecular functions, followed by 8 biological processes under low N availability (Fig 6). In leaves, the low N availability mainly upregulated DEGs associated with biological processes, metabolic and organo-nitrogen metabolic processes, while the membrane and its integral components, and protein modification processes were over-represented in DEGs that were down-regulated. In the case of high N treatment, among the up-regulated DEGs, functional annotation showed 15 biological processes, 12 cellular components, and 3 molecular functions were significantly affected (Fig 7). While among the downregulated DEGs, 7 biological processes and cellular components, and 16 molecular functions were over-represented. Similar to the upregulated DEGs associated with biosynthetic processes in leaves under low N, high N treatment clearly represented processes related to the biosynthesis and metabolism of nitrogenous compounds and peptides. Among the downregulated DEGs, functions associated with membrane modifications, response to a stimulus, protein modifications, and transferase activity were significantly over-represented in leaf tissue in high N treatment.

Overall, in root tissue, the GO terms such as membrane and cellular components, biological processes associated with a single organism, response to stimulus and transferase activity were over-represented in the up-regulated DEGs while nutrient reservoir activity related function was represented in the down-regulated DEGs due to N stress (S5 Fig). While in the case of leaf tissue, only the functions associated with catalytic, transferase, and kinase activities were represented in the up-regulated DEGs during N stress (S6 Fig). Taken together, N stress over-represented GO terms such as biological processes, molecular functions like catalytic and transferase activity, and cellular locations like a membrane or its components in the up-regulated DEGs (S7 Fig).

### KEGG pathways associated with differentially expressed genes

**Fig 8:**
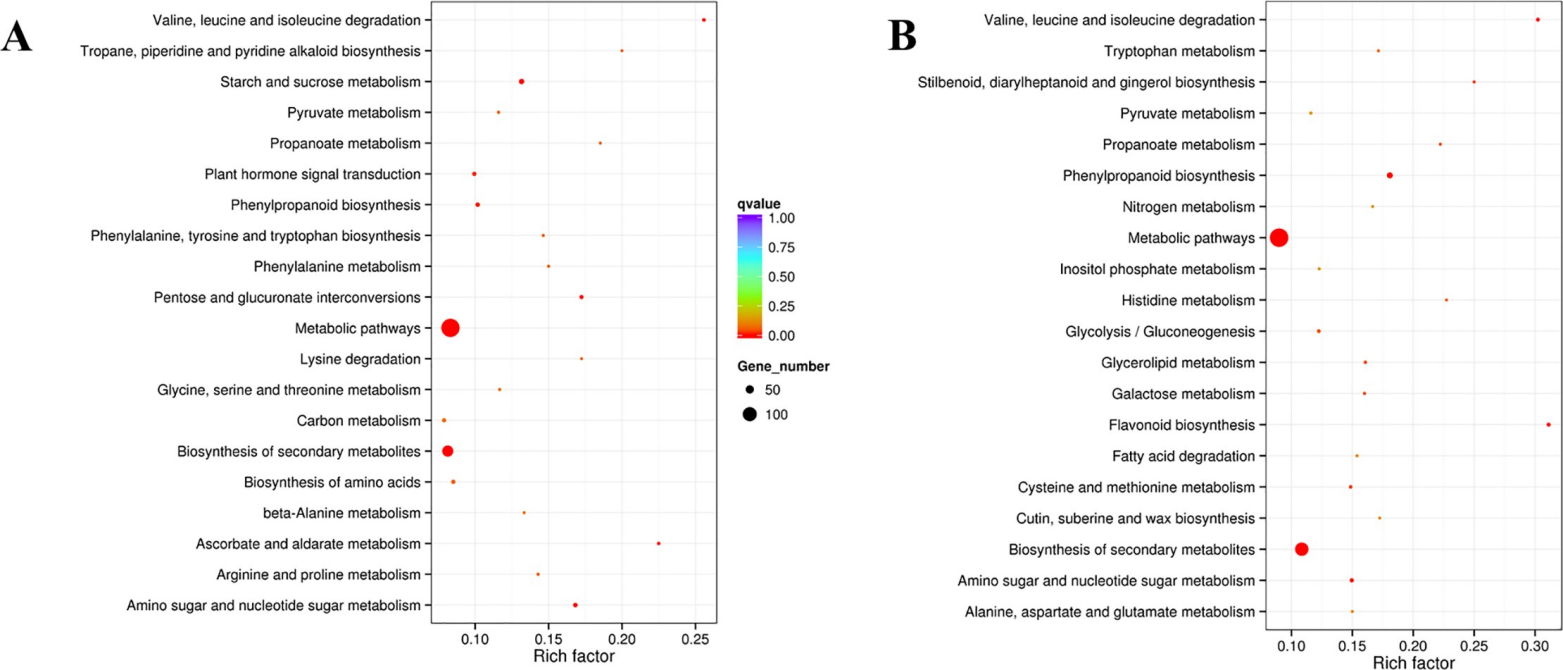
KEGG pathway enrichment scatters plot analysis of DEGs in leaf (A) and root (B) tissues between the N treatments. The Rich factor is the ratio of DEGs in this pathway term to all gene numbers annotated in this pathway term. A q value is the corrected p-value ranging from 0 to 1, and a lower value indicates higher pathway enrichment. The pathway names are shown on the vertical axis, rich factor on the horizontal axis, the size of the point represents the number of DEGs, and the color of the dot represents the q value.

The enrichment of KEGG pathways was similar in leaf and root tissues in both N treatments (Fig 8), showing over-representation of DEGs associated with metabolic pathways, especially in amino acid metabolism and biosynthesis of secondary metabolites. DEGs related to amino acid metabolic pathways such as Ala, Asp, and Glu synthesis, glutathione metabolism, phenylalanine metabolism were enriched in both N treatments. KEGG pathways of DEGs identified in both N treatments in leaf Vs. root tishsue is shown in S8 Fig. Like tissue types, the analysis showed that the number of DEGs in both N treatments were also associated with metabolic and biosynthesis of secondary metabolites pathways.

### Quantitative real-time-PCR validation of DEGs from RNA-Seq

To validate RNA-seq results, we selected twenty DEGs to confirm their expression patterns of the by quantitative real-time PCR. The fold-changes of the selected genes using RT-qPCR were consistent (S9 Fig) with the results obtained with RNA-Seq analysis (R^2^ = 0.085, r^2^ = 0.93 for roots and R^2^ = 0.080, r^2^ = 0.91 for leaf), indicating reproducibility and credibility of the RNA-seq data to evaluate the expression of nitrogen-responsive genes in spinach. Histograms were produced by comparing the Log2 fold-changes by transcriptome analysis and RT-qPCR (S10 Fig).

### Key genes responding to N perturbations

A complex network of developmental, physiological, biochemical, and molecular responses regulates uptake and partitioning of N. We selected a subset of DEGs that were directly associated with N metabolisms, such as N uptake, assimilation, and transport to understand their role in leaf or root tissues based on N availability.

### Nitrate transporters

Among the four families of transporters, Nitrate Transporter 1 (NRT1) [42] and Nitrate Transporter 2 (NRT2) [43] participate in NO_3_^−^ uptake, distribution, or storage. As per the external NO_3_^−^ concentration, plants use two specialized transport systems [44], a constitutive, low-affinity transport system (LATS) and an inducible, high-affinity transport system (HATS) to maximize N uptake efficiency [45–47]. The LATS allows transport during high external NO_3_^−^ while low concentrations activate HATS [44], although it also contributes to fulfilling NO_3_^−^ demand at higher concentrations [48]. We first looked at differentially expressed NO_3_^−^ transporters under HATS induced in roots in response to N stress. We found at least three *NRT2*.*1* (*Spo03988*, *Spo09968*, *Spo03990*) members from HATS were induced in roots due to N stress. In particular, the expression of *Spo03988* (*NRT2*.*5*) and *Spo09968* (*NRT2*.*1*) were upregulated 4.3 and 2.6-fold higher in N stressed roots. The affinity of NRT2 transporters for now NO_3_^−^ influx in the root has already been demonstrated for NRT2.1 and NRT2.5 [43, 49, 50]. NRT2.5 is a part of HATS that enables roots to absorb NO_3_^−^ under limited availability, contributes to phloem loading in shoots, and activates nitrate-inducible genes [49, 51]. Both; *NRT2*.*1* and *NRT2*.*5* in *Arabidopsis* have been characterized as NO_3_^−^ responsive genes [52]. Additionally, expression of *Spo15883* (*NRG2*, *Nitrate regulatory gene*) was also induced in roots due to limited N availability. These genes are required for NO_3_^−^ signaling and regulate expression of the nitrate-responsive genes, including NRT2.1 [53]. Under limited N, we also observed the induction of three *ammonium transporters* (*Spo24676*, *Spo16971*, *Spo24677*), implying alternative strategies deployed by roots to accomplish N requirement. Under high N (S3 Table), the expression of *NRT1* transporters (*Spo05548*, *Spo25744*, *Spo07601*) was significantly upregulated in roots. Protein NRT1/ PTR FAMILY 6.2 has been shown to function as a low-affinity proton-dependent NO_3_^−^ transporter [54], justifying its induction in spinach roots. While NPF2.13 (NRT1/ PTR FAMILY 2.13) is involved in phloem loading and NO_3_^−^ remobilization [55]. It has been proposed that as the higher NO_3_^−^ concentrations in soil are first encountered by the advancing root tip, root tip specific NRT1 transporters would best capture the available NO_3_^−^ [56]. Intriguingly, in leaf tissue, expression of six members of the NRT1 family (*Spo17357*, *Spo25690*, *Spo01109*, *Spo19480*, *Spo20248*, *and Spo26413*) under low N and two members (*Spo24247* and *Spo25782*) under high N were significantly upregulated. It has been demonstrated that several NO_3_^−^ transporters identified in leaves are closely correlated with, e.g., stomatal opening [57], NO_3_^−^ reductase activity [52], accumulation and remobilization of NO_3_^−^ [55].

### Amino acid metabolism

In plants, inorganic N (NO_3_^−^ and NH_4_^+^) is incorporated into Glu and Gln in roots before converting to other amino acids and nitrogenous compounds. Amino acids are later transported to the sink organs by the long-distance transport systems of the plant. We found that the expression of a putative *glutamine amidotransferase GAT1* (*Spo00216*) was almost 5 -fold (Log2) up in roots as well as 4 -fold up in leaf under high N. Root specific GAT1 gene has been shown to be highly responsive to N status in *Arabidopsis* [58]. It is plausible to assume that under high N conditions, GAT1 facilitates the conversion of Gln to Glu for channeling C to the TCA cycle. We found two *Glutamate receptor* (*GLR*) genes (*Spo15921* and *Spo01837*) up-regulated in response to high N in roots. GLR genes are implicated in C: N signaling, hypocotyl elongation, root growth, stress responses, and general ion transport [59–62]. Asn and Gln serve as the major N transport and storage compounds in most non-leguminous plants [63]. The expression of *Asparagine synthetases* (*AS*) (*Spo05295* and *Spo08792*) were significantly up-regulated in the roots, and leaf under high N. AS activity is regulated by N status [60, 64] as it catalyzes the conversion of Asp and Gln to Asn and Glu in an ATP-dependent reaction. Aminotransferases have been suggested to play essential roles in redirecting N resources to different pathways [65]. The expression of the branched-chain amino acid (BCAA) aminotransferase genes *BCAT-2* (*Spo14673 and Spo13997*) regulating conversion of leucine/valine/isoleucine to Glu were significantly upregulated in roots. Among the most enriched pathway terms, several DEGs associated with degradation of branched-chain amino acids were significantly upregulated in the root (33 out of 48 reference genes annotated to the BCAA degradation pathway) as well as leaf (23 out of 48) tissues. Although BCAA catabolism likely has many functions in plants [66–68], its involvement in N redistribution needs further functional characterization of the genes involved in BCAA catabolism. Additionally, we found expression of *serine decarboxylase* (*SDC; Spo13254*) significantly upregulated in response to high N in roots. Most recently, SDC like gene showing remarkably high expression in young roots under sufficient N has been characterized in tea plants [69]. We performed a BLASTP homology search of the NCBI database. We found that the deduced protein sequence of the spinach SDC gene shared high similarity with characterized Tea *Camellia sinensis* (74.6% identity) SDC as well as other plant species such as *Arabidopsis* (77.26% identity), and rice (74.7% identity) (S11 Fig). Decarboxylation of Ser is the major source of ethanolamine production required for plant growth [70]. Moreover, it has been proposed that Ser could serve as a source of N in non-photosynthetic tissues like roots [71]. Nevertheless, additional functional characterization of this gene will be required to understand its role in N metabolism. Under high N, expression of putative amino acid transporters such as *WAT-1 related* (*UMAMIT34*, *Usually Multiple Acids Move In and out Transporters; Spo14333*), *vacuolar amino acid transporters* (*Spo21518* and *Spo21518*) and *ABC transporter C family member* (*Spo05865*) were significantly upregulated (≥2 -fold (Log2) in leaf implying increased N assimilation.

### Nucleic acid metabolism

The development and proliferation of living cells require DNA replication, which is responsible for genome duplication in plants [72]. DNA replication is initiated and facilitated by the formation of the replication fork comprising the Minichromosome Maintenance MCM(2–7) protein helicase complex [73]. The MCMs are found to be highly expressed in dividing tissues such as shoot apex and root tips of several plants [74], and their expression is coordinated during plant development, possibly at the level of transcription [75]. Consistent with these reports, nearly half of the DEGs (24 out of 50; P < 0.00029) associated with DNA replication fork assembly were up-regulated in leaf tissue under high N. Since DNA replication is linked to cell cycle progression and DNA repair processes, upregulation of genes associated with MCM complex, RFAs, genes regulating DNA polymerase complexes (α primase, δ, and ε), DNA ligases (Lig 1, Fen1) and helicase (Dna2) were not unexpected (S12 Fig). Besides replication fork, upregulation of several DEGs associated with pyrimidine (34 out 116 reference genes annotated for the pyrimidine pathway; P <0.004; S13 Fig) and purine metabolism (41 out of 158 reference genes annotated for the purine pathway; P <0.006; S14 Fig) also supported increased nucleic acid synthesis and plant growth in response to N availability.

### Hormonal pathways

Several studies have demonstrated that NO_3_^−^ can regulate biosynthesis, degradation, transport, and signaling of phytohormones [76, 77]. The auxin transport and signaling components are critical for altering root architecture in response to N availability in plants [78, 79]. A relationship between the availability of NO_3_^−^ and auxin metabolism has been validated in *Arabidopsis*, maize, and rice [80–82]. Among the DEGs associated with hormonal pathways, 11 genes coding *Auxin-binding proteins* (ABP) with putative auxin receptor function were up-regulated in roots under high N treatment. Although these proteins share homology with many ABP19 and ABP20 proteins from plants, a detailed functional characterization would be required to understand their significance during excess N availability. The Auxin Binding Protein 1 (ABP1) has been extensively studied as a candidate auxin receptor and has been shown to be a key regulator for auxin-mediated responses [83, 84]. Due to N stress, two *GA2oxs*; *Gibberellin 2-beta-dioxygenase* (*Spo11903* and *Spo03697*) and a *Gibberellin-regulated* (*Spo07806*) genes were up-regulated in roots. *GA2oxs* regulate plant growth by inactivating endogenous bioactive gibberellins. The gibberellin's impact on leaf‐growth and enhanced apical dominance is well known; however, little information is available about root-specific changes in gibberellin in response to N stress. Nonetheless, reduction in endogenous GA concentrations in roots due to the down-regulation of GA biosynthesis at the transcriptional level has been shown to regulate root growth and NO_3_^−^ uptake in cucumber plants [85].

### Carbon metabolism

In high N treatment, several genes associated with C metabolism were differentially expressed in leaf tissue. In particular, the expression of *Phosphoenolpyruvate carboxykinase* (*PEPCK; Spo20411*) was up-regulated by 4-fold (Log2 scale). PEPCK is involved in multiple C metabolism-related functions such as CO_2_ assimilation, relocating the consumed TCA intermediates in the biosynthesis, and assimilation of N [86]. Similarly, expression of *Isocitrate lyase* (*ICL*; Spo13898) and *Malate synthase* (*MS*; Spo16696) involved in the glyoxylate pathway were up-regulated (>2-fold Log2). It has been proposed that cellular lipids can be metabolized via the glyoxylate cycle. Sucrose can be synthesized from the four C products of the glyoxylate cycle, which converts TCA cycle components to sucrose *via* the process of gluconeogenesis. Although detailed enzyme kinetics and tracer experiments will be required to understand the role of these genes in spinach, unusually coordinated expression of *PEPCK* with *MS* and *ICL* poses a possibility of the presence of gluconeogenic pathway in leaf tissue. In C4 plants, like maize, gluconeogenesis has been shown to regulate the import/mobilization of nitrogenous compounds. Up-regulation of *Expansins* (*Spo03956* and *Spo11774*) and *Sugar transporter* proteins (*MST4; Spo11108* and *Spo11108*) and *Beta-amylase 2* (*Spo10422*) also supports cell expansion and enhanced influx of photo-assimilates in leaf tissue under high N treatment, respectively.

### Differentially expressed transcription factors (TFs) responding to N stress

**Fig 9:**
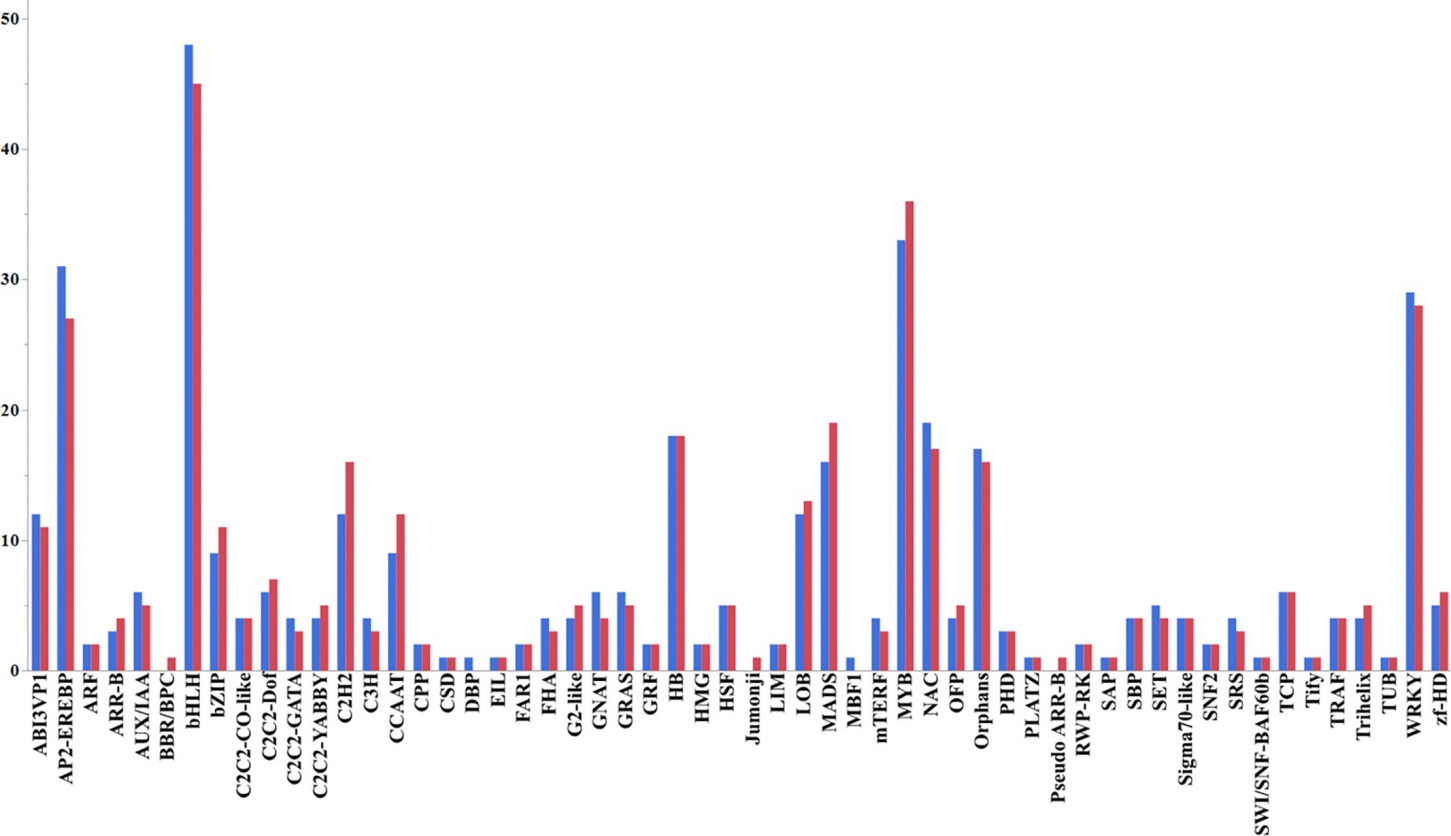
Transcription factor analysis. The graph shows the number of transcription factors (vertical axis) from 56 TF families that expressed genes differentially (change in the expression <2 Log2-fold) in leaf tissue under Low-N or High-N. iTAK is used to perform the transcription factor analysis of plants (p-value < 0.05). The details of TFs families are published in Zheng et al., 2016.

We used iTAK [87] to perform the transcription factor analysis. A total of 786 transcription factor related genes were identified from spinach and the 56 TF families that expressed genes differentially between leaf Vs. root tissues under low N or high N are shown in Fig 9. The largest members of the TFs belonged to the bHLH family (48), followed by *MYB* (33), *AP2-EREBP* (31), *WRKY* (29), *NAC* (19), in case of high N treatment. While for low N, the bHLH family (45), followed by *MYB* (36), *AP2-EREBP* (27), *WRKY* (28), *NAC* (17) members of the families, were differentially regulated. The distribution of the differentially expressed TFs between leaf vs. root tissues under the N stress was separately investigated. A list of differentially expressed TFs showing at least ≥ 2 Log2 fold significant (p-value < 0.05) change in the expression in leaf and root tissues due to N treatments (S7 Table). It will be valuable to functionally characterize the identified TFs for enhancing plant performance under limited N availability and thereby providing novel targets for genetic manipulation.

In roots, low N up-regulated expression (≥ 2-fold log2) of three *ERFs—TINY* (*Spo16233*), *ERF17* (Spo20470), and *ERF12* (*Spo10826*); and two NAC TFs (*NAC43; Spo25926* and *NAC86; Spo15445*). The interaction between ethylene and availability of N affects numerous physiological processes, including roots architecture, leaf, reproductive organ development, as well as the synthesis of amino acids, proteins, and enzymes [88]. N starvation increases the number or affinity of root receptors, allowing roots to respond to lower concentrations of ethylene than those found in unstressed roots. The AP2/ERF-TF family has been involved in signaling processes and the responses to environmental stresses [89], such as micronutrient deficiency [90, 91]. Similar to our observations, under N starvation, seven ERF genes in cucumber seedlings were also differentially regulated [92]. Under high N, the expression of *Ethylene-responsive transcription factor 13* (*ERF13; Spo11940*) and two of the *NAC29* TFs was induced (≥ 2-fold Log2). We found that the expression of *1-aminocyclopropane-1-carboxylate oxidase1* (*ACO*; *Spo03782* and *Spo03777*) genes involved in ethylene synthesis were also upregulated in roots. Induction of expression and enzymatic activities of ethylene biosynthetic genes, *ACO*, and *ACC synthase* (*ACS*) in response to high N has been demonstrated in other plants [93–95].

## Conclusions

In conclusion, comparative analysis of the leaf and root transcriptomes indicated a coordinated regulation of multiple genes and pathways and provided a catalog of transcriptomic variation between the spinach tissues in response to N. Although transcriptomic responses to N perturbation have been studied in Arabidopsis and few other crops, to the best of our knowledge, this is the first study that provides information about the partitioning of leaf and root transcriptomic responses in spinach. The genomic resources generated from this RNA-Seq experiment can be used to characterize genes associated with NUE in spinach breeding materials and to develop informative markers specifically to select parental lines. RNA-seq analysis revealed that N stress caused most transcriptomic changes in roots, identifying 1,346 DEGs compared to 1136 DEGs in leaf. Significant upregulation of *NRT2*.*1* (*Spo03988*, *Spo09968*, *Spo03990*) in roots validated its role in N acquisition under limited availability. The root-specific genetic manipulation to alter the expression of *NRT2*.*1* could be a way forward to enhance NUE in spinach. Concomitant activation of putative *glutamine amidotransferase GAT1* (Spo00216) and *Asparagine synthetases* (AS) (Spo05295 and Spo08792) along with *Glutamate receptor* (*GLR*) (Spo15921 and Spo01837) in response to excess N highlights the functional significance of Gln reduction in N assimilation. Consistent with the effects of higher N availability on plant growth, expression of genes associated with nucleic acid synthesis and DNA replication were significantly upregulated. The RNA-Seq data generated in this study will be a valuable resource for the identification of key genes related to N metabolism and identifying potential targets for genetic manipulation to enhance N uptake and utilization. The functional characterization of the differentially expressed genes during N stress would provide new insights into developing N use efficient varieties.

## Supporting information

S1 Fig Heat maps of the correlation coefficient between samples. The scatter diagrams demonstrate the correlation coefficient between all samples, R2, the square of the Pearson coefficient. LNR: low N root; LNL: low N leaf; HNR: high N root and HNL: high N leaf; The numbers 1,2,3 are independent replications.
(TIF)

S2 Fig Different gene expression levels in N treatments. FPKM violin Plot, the x-axis shows the sample names where T2 is high N and T1 low N; LF = Leaf and RT = Root, and the y-axis shows the log10(FPKM+1). Each violin has five statistical magnitudes (max value, upper quartile, median, lower quartile, and min value). The violin width shows the gene density.
(TIF)

S3 Fig Hierarchical cluster analysis of differential gene expression in the leaf and root of spinach plants at high N and low N. (TIF)

S4 Fig The volcano maps showing the number of differentially expressed genes in spinach plants grown under High-N. Red dots represent up-regulated genes, and green dots represent down-regulated genes (p < 0.05).
(TIF)

S5 Fig **Gene ontology (GO) enrichment bar chart of DEGs showing most enriched GO terms of (A) upregulated and (B) downregulated DEGs in root tissue under Low-N.** The y-axis is the enriched GO term, and the x-axis is the number of DEGs enriched in the listed term. Colors represent different GO types: biological process, cellular component, and molecular function. * significantly enriched term (p-value < 0.05).
(TIF)

S6 Fig **Gene ontology (GO) enrichment bar chart of DEGs showing most enriched GO terms of (A) upregulated and (B) downregulated DEGs in leaf tissue under low N.** The y-axis is the enriched GO term, the x-axis is the number of DEGs enriched in the listed term. Colors represent different GO types: biological process, cellular component, and molecular function. * significantly enriched term (p-value < 0.05).
(TIF)

S7 Fig Gene ontology (GO) enrichment bar chart of DEGs showing most enriched GO terms of (A) upregulated and (B) downregulated DEGs in Low-N in both tissues. The y-axis is the enriched GO term, and the x-axis is the number of DEGs enriched in the listed term. Colors represent different GO types: biological process, cellular component, and molecular function. * significantly enriched term (p-value < 0.05)
(TIF)

S8 Fig **KEGG pathway enrichment scatter-plot analysis of DEGs in low N (A) and high N (B) in leaf Vs. root tissue.** The Rich factor is the ratio of DEGs in this pathway term to all gene numbers annotated in this pathway term. A q value is the corrected p-value ranging from 0 to 1, and a lower value indicates greater pathway enrichment. The pathway names are shown on the vertical axis, rich factor on the horizontal axis, the size of the point represents the number of DEGs, and the color of the dot represents the q value
(TIF)

S9 Fig Linear regressions involving the RNA sequencing data and the RT-qPCR validation data expressed in terms of Log2 fold-change (FC). FC was calculated as the ratio between the drought-stressed and control plants. (A, B) indicate roots and leaves, respectively. * Significant Pearson’s correlation coefficient P < 0.01. (R2 = 0.85 and 0.80 for root and leaf tissues respectively).
(TIF)

S10 Fig qRT–PCR validation of RNA-seq results. Twenty randomly selected DEGs identified by RNA-seq (red bars) in roots, and leaf tissue of spinach were selected to analyze by qRT-PCR (black bars). Comparison of the fold change of RNA-seq and qRT–PCR shows a correlation coefficient of 0.94, indicating the reliability of RNA-seq results. Error bars represent the SEM.
(TIF)

S11 Fig CLUSTAL W (1.83) multiple sequence alignment of spinach SDC. Multiple alignments of the protein sequence of spinach SDC (Spo13254) with selected SDCs from other species. Identical amino acids are shown with asterisk *. The NCBI GeneBank IDs used to Serine decarboxylases from plants are as follows: *Arabidopsis* (NP_175036.1), *Camellia sinensis* (XP_028123129.1), *Solanum lycopersicum* (XP_004237774.1), *Oryza sativa Japonica* Group (BAS79103.1), Zea mays (PWZ22363.1), *Spinacia oleracea* (Spo13254)
(TIFF)

S12 Fig DNA replication fork (KEGG: 03030). DNA replication pathway diagram highlighting DEGs up-regulated (green boxes with red border) in leaf tissue under high N.
(TIF)

S13 Fig Pyrimidine metabolism (KEGG: 00240). Diagram highlighting DEGs up-regulated (green boxes with red border) in leaf tissue under high N.
(TIF)

S14 Fig Purine metabolism (KEGG: 00230). Diagram highlighting DEGs up-regulated (green boxes with red border) in leaf tissue under high N.
(TIF)

S1 Table Primers used for RT-qPCR. (XLSX)

S2 Table Detail statistics of sequencing results. Total mapped—number of reads that can be mapped to the reference genome; Multiple mapped—number of reads mapped to multiple sites in the reference genome. Uniquely mapped—number of reads mapped to the reference genome; the number of reads mapped to the positive strand (+) or the minus strand (-); Splice reads mapped to two exons (junction reads), and non-splice reads are mapped entirely to a single exon.HN = high N; LN = low N, L1, L2 L3 = Leaf replications 1, 2,3; R1, R2, R3 = Root Replications 1,2,3.
(XLSX)

S3 Table List of differentially expressed genes upregulated in roots under high N treatment. (XLSX)

S4 Table List of differentially expressed genes upregulated in roots under low N treatment. (XLSX)

S5 Table List of differentially expressed genes upregulated in leaf under high N treatment. (XLSX)

S6 Table List of differentially expressed genes upregulated in leaf under low N treatment. (XLSX)

S7 Table Differentially expressed transcription factors (TFs). TFs with <2-fold change (Log2) in the expression due to N stress roots and leaf tissues (P-value < 0.05). The details of TFs families are published [87].
(XLSX)

## References

[1] Crawford NM. Nitrate: nutrient and signal for plant growth. Plant Cell. 1995;7(7):859–68. 10.1105/tpc.7.7.859 7640524 PMC160877

[2] Stitt M, Müller C, Matt P, Gibon Y, Carillo P, Morcuende R, et al Steps towards an integrated view of nitrogen metabolism. Journal of Experimental Botany. 2002;53(370):959–70. 10.1093/jexbot/53.370.959 11912238

[3] Levin SA, Mooney H, Field C. The dependence of plant root: shoot ratios on internal nitrogen concentration. Annals of Botany. 1989;64(1):71–5.

[4] Hermans C, Hammond JP, White PJ, Verbruggen N. How do plants respond to nutrient shortage by biomass allocation? Trends in plant science. 2006;11(12):610–7. 10.1016/j.tplants.2006.10.007 17092760

[5] Smolders E, Merckx R. Growth and shoot: root partitioning of spinach plants as affected by nitrogen supply. Plant, Cell & Environment. 1992;15(7):795–807.

[6] Chan-Navarrete R, Kawai A, Dolstra O, van Bueren ETL, van der Linden CG. Genetic diversity for nitrogen use efficiency in spinach (Spinacia oleracea L.) cultivars using the Ingestad model on hydroponics. Euphytica. 2014;199(1–2):155–66.

[7] Mulvaney RL, Khan S, Ellsworth T. Synthetic nitrogen fertilizers deplete soil nitrogen: a global dilemma for sustainable cereal production. Journal of environmental quality. 2009;38(6):2295–314. 10.2134/jeq2008.0527 19875786

[8] Socolow RH. Nitrogen management and the future of food: lessons from the management of energy and carbon. Proceedings of the National Academy of Sciences. 1999;96(11):6001–8. 10.1073/pnas.96.11.6001 PMC34219 10339531

[9] Marvi MSP. Effect of nitrogen and phosphorous rates on fertilizer use efficiency in lettuce and spinach. Journal of Horticulture and Forestry. 2009;1(7):140–7.

[10] Schenk M, Heins B, Steingrobe B. The significance of root development of spinach and kohlrabi for N fertilization. Plant and Soil. 1991;135(2):197–203.

[11] Neeteson J, Carton O, editors. The environmental impact of nitrogen in field vegetable production. International Conference on Environmental Problems Associated with Nitrogen Fertilisation of Field Grown Vegetable Crops 563; 1999.

[12] Stagnari F, Di Bitetto V, Pisante M. Effects of N fertilizers and rates on yield, safety and nutrients in processing spinach genotypes. Scientia Horticulturae. 2007;114(4):225–33.

[13] Maynard D, Barker A, Minotti P, Peck N. Nitrate accumulation in vegetables. Advances in Agronomy. 28: Elsevier; 1976 p. 71–118.

[14] Hirel B, Le Gouis J, Ney B, Gallais A. The challenge of improving nitrogen use efficiency in crop plants: towards a more central role for genetic variability and quantitative genetics within integrated approaches. Journal of experimental botany. 2007;58(9):2369–87. 10.1093/jxb/erm097 17556767

[15] Bi YM, Zhang Y, Signorelli T, Zhao R, Zhu T, Rothstein S. Genetic analysis of Arabidopsis GATA transcription factor gene family reveals a nitrate‐inducible member important for chlorophyll synthesis and glucose sensitivity. The Plant Journal. 2005;44(4):680–92. 10.1111/j.1365-313X.2005.02568.x 16262716

[16] Zhang H, Forde BG. An Arabidopsis MADS box gene that controls nutrient-induced changes in root architecture. Science. 1998;279(5349):407–9. 10.1126/science.279.5349.407 9430595

[17] Watanabe CK, Hachiya T, Takahara K, Kawai-Yamada M, Uchimiya H, Uesono Y, et al Effects of AOX1a deficiency on plant growth, gene expression of respiratory components and metabolic profile under low-nitrogen stress in Arabidopsis thaliana. Plant and cell physiology. 2010;51(5):810–22. 10.1093/pcp/pcq033 20304787

[18] Beatty PH, Klein MS, Fischer JJ, Lewis IA, Muench DG, Good AG. Understanding Plant Nitrogen Metabolism through Metabolomics and Computational Approaches. Plants. 2016;5(4):39. 10.3390/plants5040039 PMC5198099 27735856

[19] Perchlik M, Tegeder M. Improving plant nitrogen use efficiency through alteration of amino acid transport processes. Plant physiology. 2017;175(1):235–47. 10.1104/pp.17.00608 28733388 PMC5580756

[20] Wang R, Okamoto M, Xing X, Crawford NM. Microarray analysis of the nitrate response in Arabidopsis roots and shoots reveals over 1,000 rapidly responding genes and new linkages to glucose, trehalose-6-phosphate, iron, and sulfate metabolism. Plant physiology. 2003;132(2):556–67. 10.1104/pp.103.021253 12805587 PMC166997

[21] Peng M, Bi Y-M, Zhu T, Rothstein SJ. Genome-wide analysis of Arabidopsis responsive transcriptome to nitrogen limitation and its regulation by the ubiquitin ligase gene NLA. Plant molecular biology. 2007;65(6):775–97. 10.1007/s11103-007-9241-0 17885809

[22] Palenchar PM, Kouranov A, Lejay LV, Coruzzi GM. Genome-wide patterns of carbon and nitrogen regulation of gene expression validate the combined carbon and nitrogen (CN)-signaling hypothesis in plants. Genome biology. 2004;5(11):R91 10.1186/gb-2004-5-11-r91 15535867 PMC545782

[23] Lian X, Wang S, Zhang J, Feng Q, Zhang L, Fan D, et al Expression profiles of 10,422 genes at early stage of low nitrogen stress in rice assayed using a cDNA microarray. Plant molecular biology. 2006;60(5):617–31. 10.1007/s11103-005-5441-7 16649102

[24] Cai H, Lu Y, Xie W, Zhu T, Lian X. Transcriptome response to nitrogen starvation in rice. Journal of biosciences. 2012;37(4):731–47. 10.1007/s12038-012-9242-2 22922198

[25] Yang W, Yoon J, Choi H, Fan Y, Chen R, An G. Transcriptome analysis of nitrogen-starvation-responsive genes in rice. BMC Plant Biology. 2015;15(1):31. 25644226 10.1186/s12870-015-0425-5 PMC4333837

[26] Zamboni A, Astolfi S, Zuchi S, Pii Y, Guardini K, Tononi P, et al Nitrate induction triggers different transcriptional changes in a high and a low nitrogen use efficiency maize inbred line. Journal of integrative plant biology. 2014;56(11):1080–94. 10.1111/jipb.12214 24805158

[27] Humbert S, Subedi S, Cohn J, Zeng B, Bi Y-M, Chen X, et al Genome-wide expression profiling of maize in response to individual and combined water and nitrogen stresses. BMC genomics. 2013;14(1):3. 23324127 10.1186/1471-2164-14-3 PMC3571967

[28] Wang J, Song K, Sun L, Qin Q, Sun Y, Pan J, et al Morphological and Transcriptome Analysis of Wheat Seedlings Response to Low Nitrogen Stress. Plants. 2019;8(4):98. 10.3390/plants8040098 PMC6524375 30991719

[29] Curci PL, Cigliano RA, Zuluaga DL, Janni M, Sanseverino W, Sonnante G. Transcriptomic response of durum wheat to nitrogen starvation. Scientific reports. 2017;7(1):1176 10.1038/s41598-017-01377-0 28446759 PMC5430780

[30] Goel P, Sharma NK, Bhuria M, Sharma V, Chauhan R, Pathania S, et al Transcriptome and Co-Expression Network Analyses Identify Key Genes Regulating Nitrogen Use Efficiency in Brassica juncea L. Scientific Reports. 2018;8(1):7451 10.1038/s41598-018-25826-6 29748645 PMC5945678

[31] Canali S, Montemurro F, Tittarelli F, Masetti O. Effect of nitrogen fertilisation reduction on yield, quality and N utilisation of processing spinach. Journal of food, agriculture & environment. 2008;6(3&4):242–7.

[32] Canali S, Montemurro F, Tittarelli F, Masetti O. Is it possible to reduce nitrogen fertilization in processing spinach? Journal of plant nutrition. 2011;34(4):534–46.

[33] Chan-Navarrete R, Dolstra O, van Kaauwen M, van Bueren ETL, van der Linden CG. Genetic map construction and QTL analysis of nitrogen use efficiency in spinach (Spinacia oleracea L.). Euphytica. 2016;208(3):621–36.

[34] Liu M, Liu X, Ding W, Chen Q, Lin X. Variation in nitrogen uptake and utilization efficiency in spinach genotypes and its evaluation. Journal of Zhejiang University (Agriculture and Life Sciences). 2012;38(5):599–607.

[35] Joshi V, Joshi M, Silwal D, Noonan K, Rodriguez S, Penalosa A. Systematized biosynthesis and catabolism regulate citrulline accumulation in watermelon. Phytochemistry. 2019;162:129–40. 10.1016/j.phytochem.2019.03.003 30884257

[36] Trapnell C, Williams BA, Pertea G, Mortazavi A, Kwan G, van Baren MJ, et al Transcript assembly and quantification by RNA-Seq reveals unannotated transcripts and isoform switching during cell differentiation. Nat Biotechnol. 2010;28(5):511–5. 10.1038/nbt.1621 20436464 PMC3146043

[37] Anders S, Huber W. Differential expression analysis for sequence count data. Genome Biology. 2010;11(10):R106 10.1186/gb-2010-11-10-r106 20979621 PMC3218662

[38] Ashburner M, Ball CA, Blake JA, Botstein D, Butler H, Cherry JM, et al Gene Ontology: tool for the unification of biology. Nat Genet. 2000;25(1):25–9. 10.1038/75556 10802651 PMC3037419

[39] Kanehisa M, Goto S, Kawashima S, Okuno Y, Hattori M. The KEGG resource for deciphering the genome. Nucleic Acids Research. 2004;32(suppl_1):D277–D80. 14681412 10.1093/nar/gkh063 PMC308797

[40] Yan J, Yu L, Xuan J, Lu Y, Lu S, Zhu W. De novo transcriptome sequencing and gene expression profiling of spinach (Spinacia oleracea L.) leaves under heat stress. Scientific reports. 2016;6:19473 10.1038/srep19473 26857466 PMC4746569

[41] Pfaffl MW. A new mathematical model for relative quantification in real-time RT–PCR. Nucleic acids research. 2001;29(9):e45–e. 10.1093/nar/29.9.e45 11328886 PMC55695

[42] Léran S, Varala K, Boyer J-C, Chiurazzi M, Crawford N, Daniel-Vedele F, et al A unified nomenclature of NITRATE TRANSPORTER 1/PEPTIDE TRANSPORTER family members in plants. Trends in plant science. 2014;19(1):5–9. 10.1016/j.tplants.2013.08.008 24055139

[43] Orsel M, Krapp A, Daniel-Vedele F. Analysis of the NRT2 nitrate transporter family in Arabidopsis. Structure and gene expression. Plant physiology. 2002;129(2):886–96. 10.1104/pp.005280 12068127 PMC161709

[44] Miller AJ, Fan X, Orsel M, Smith SJ, Wells DM. Nitrate transport and signalling. Journal of experimental Botany. 2007;58(9):2297–306. 10.1093/jxb/erm066 17519352

[45] Aslam M, Travis RL, Huffaker RC. Comparative kinetics and reciprocal inhibition of nitrate and nitrite uptake in roots of uninduced and induced barley (Hordeum vulgare L.) seedlings. Plant Physiology. 1992;99(3):1124–33. 11537883 10.1104/pp.99.3.1124 PMC1080592

[46] Behl R, Tischner R, Raschke K. Induction of a high-capacity nitrate-uptake mechanism in barley roots prompted by nitrate uptake through a constitutive low-capacity mechanism. Planta. 1988;176(2):235–40. 10.1007/BF00392450 24220778

[47] Little DY, Rao H, Oliva S, Daniel-Vedele F, Krapp A, Malamy JE. The putative high-affinity nitrate transporter NRT2. 1 represses lateral root initiation in response to nutritional cues. Proceedings of the National Academy of Sciences. 2005;102(38):13693–8. 10.1073/pnas.0504219102 PMC1224627 16157886

[48] Malagoli P, Lainé P, Le Deunff E, Rossato L, Ney B, Ourry A. Modeling Nitrogen Uptake in Oilseed Rape cv Capitol during a Growth Cycle Using Influx Kinetics of Root Nitrate Transport Systems and Field Experimental Data. Plant Physiology. 2004;134(1):388–400. 10.1104/pp.103.029538 14671012 PMC316318

[49] Lezhneva L, Kiba T, Feria‐Bourrellier AB, Lafouge F, Boutet‐Mercey S, Zoufan P, et al The Arabidopsis nitrate transporter NRT 2.5 plays a role in nitrate acquisition and remobilization in nitrogen‐starved plants. The Plant Journal. 2014;80(2):230–41. 10.1111/tpj.12626 25065551

[50] Kiba T, Krapp A. Plant nitrogen acquisition under low availability: regulation of uptake and root architecture. Plant and Cell Physiology. 2016;57(4):707–14. 10.1093/pcp/pcw052 27025887 PMC4836452

[51] Kotur Z, Glass AD. A 150 kDa plasma membrane complex of AtNRT2.5 and AtNAR2.1 is the major contributor to constitutive high-affinity nitrate influx in Arabidopsis thaliana. Plant Cell Environ. 2015;38(8):1490–502. 10.1111/pce.12496 25474587

[52] Loque D, Tillard P, Gojon A, Lepetit M. Gene expression of the NO3- transporter NRT1.1 and the nitrate reductase NIA1 is repressed in Arabidopsis roots by NO2-, the product of NO3- reduction. Plant Physiol. 2003;132(2):958–67. 10.1104/pp.102.018523 12805624 PMC167034

[53] Xu N, Wang R, Zhao L, Zhang C, Li Z, Lei Z, et al The Arabidopsis NRG2 Protein Mediates Nitrate Signaling and Interacts with and Regulates Key Nitrate Regulators. Plant Cell. 2016;28(2):485–504. 10.1105/tpc.15.00567 26744214 PMC4790867

[54] Chiu CC, Lin CS, Hsia AP, Su RC, Lin HL, Tsay YF. Mutation of a nitrate transporter, AtNRT1:4, results in a reduced petiole nitrate content and altered leaf development. Plant Cell Physiol. 2004;45(9):1139–48. 10.1093/pcp/pch143 15509836

[55] Fan SC, Lin CS, Hsu PK, Lin SH, Tsay YF. The Arabidopsis nitrate transporter NRT1.7, expressed in phloem, is responsible for source-to-sink remobilization of nitrate. Plant Cell. 2009;21(9):2750–61. 10.1105/tpc.109.067603 19734434 PMC2768937

[56] Garnett T, Conn V, Plett D, Conn S, Zanghellini J, Mackenzie N, et al The response of the maize nitrate transport system to nitrogen demand and supply across the lifecycle. New Phytol. 2013;198(1):82–94. 10.1111/nph.12166 23398565

[57] Guo FQ, Young J, Crawford NM. The nitrate transporter AtNRT1.1 (CHL1) functions in stomatal opening and contributes to drought susceptibility in Arabidopsis. Plant Cell. 2003;15(1):107–17. 10.1105/tpc.006312 12509525 PMC143464

[58] Zhu H, Kranz R. A Nitrogen-Regulated Glutamine Amidotransferase (GAT1_2.1) Represses Shoot Branching in Arabidopsis. Plant physiology. 2012;160. 10.1104/pp.112.199364 PMC3510109 22885937

[59] Gilliham M, Campbell M, Dubos C, Becker D, Davenport R. The Arabidopsis thaliana Glutamate-like Receptor Family (AtGLR) In: Baluška F, Mancuso S, Volkmann D, editors. Communication in Plants: Neuronal Aspects of Plant Life. Berlin, Heidelberg: Springer Berlin Heidelberg; 2006 p. 187–204.

[60] Lam HM, Chiu J, Hsieh MH, Meisel L, Oliveira IC, Shin M, et al Glutamate-receptor genes in plants. Nature. 1998;396(6707):125–6. 10.1038/24066 9823891

[61] Davenport R. Glutamate receptors in plants. Annals of botany. 2002;90(5):549–57. 10.1093/aob/mcf228 12466095 PMC4240445

[62] Price MB, Jelesko J, Okumoto S. Glutamate receptor homologs in plants: functions and evolutionary origins. Front Plant Sci. 2012;3:235–. 10.3389/fpls.2012.00235 23115559 PMC3483616

[63] Ireland R. The enzymes of glutamine, glutamate, asparagine, and aspartate metabolism. Plant Amino Acids: Biochemistry and Metabolism. 1999.

[64] Antunes F, Aguilar M, Pineda M, Sodek L. Nitrogen stress and the expression of asparagine synthetase in roots and nodules of soybean (Glycine max). Physiologia Plantarum. 2008;133(4):736–43. 10.1111/j.1399-3054.2008.01092.x 18384503

[65] Brosnan JT. Glutamate, at the interface between amino acid and carbohydrate metabolism. The Journal of nutrition. 2000;130(4):988S–90S. 10736367 10.1093/jn/130.4.988S

[66] Gonda I, Bar E, Portnoy V, Lev S, Burger J, Schaffer AA, et al Branched-chain and aromatic amino acid catabolism into aroma volatiles in Cucumis melo L. fruit. Journal of experimental botany. 2010;61(4):1111–23. 10.1093/jxb/erp390 20065117 PMC2826658

[67] Däschner K, Couée I, Binder S. The mitochondrial isovaleryl-coenzyme a dehydrogenase of arabidopsis oxidizes intermediates of leucine and valine catabolism. Plant physiology. 2001;126(2):601–12. 10.1104/pp.126.2.601 11402190 PMC111152

[68] Kochevenko A, Araújo WL, Maloney GS, Tieman DM, Do PT, Taylor MG, et al Catabolism of Branched Chain Amino Acids Supports Respiration but Not Volatile Synthesis in Tomato Fruits. Molecular Plant. 2012;5(2):366–75. 10.1093/mp/ssr108 22199237

[69] Bai P, Wei K, Wang L, Zhang F, Ruan L, Li H, et al Identification of a Novel Gene Encoding the Specialized Alanine Decarboxylase in Tea (Camellia sinensis) Plants. Molecules. 2019;24(3):540. 10.3390/molecules24030540 PMC6384637 30717241

[70] Kwon Y, Yu SI, Lee H, Yim JH, Zhu JK, Lee BH. Arabidopsis serine decarboxylase mutants implicate the roles of ethanolamine in plant growth and development. Int J Mol Sci. 2012;13(3):3176–88. 10.3390/ijms13033176 22489147 PMC3317708

[71] Hartung W, Ratcliffe RG. Utilization of glycine and serine as nitrogen sources in the roots of Zea mays and Chamaegigas intrepidus. J Exp Bot. 2002;53(379):2305–14. 10.1093/jxb/erf092 12432023

[72] Sanchez MdlP, Costas C, Sequeira-Mendes J, Gutierrez C. Regulating DNA replication in plants. Cold Spring Harb Perspect Biol. 2012;4(12):a010140 10.1101/cshperspect.a010140 23209151 PMC3504439

[73] Duderstadt KE, Reyes-Lamothe R, van Oijen AM, Sherratt DJ. Replication-fork dynamics. Cold Spring Harb Perspect Biol. 2014;6(1). 10.1101/cshperspect.a010157 PMC3941221 23881939

[74] Tuteja N, Tran NQ, Dang HQ, Tuteja R. Plant MCM proteins: role in DNA replication and beyond. Plant Molecular Biology. 2011;77(6):537–45. 10.1007/s11103-011-9836-3 22038093

[75] Shultz RW, Lee T-J, Allen GC, Thompson WF, Hanley-Bowdoin L. Dynamic localization of the DNA replication proteins MCM5 and MCM7 in plants. Plant physiology. 2009;150(2):658–69. 10.1104/pp.109.136614 19357199 PMC2689970

[76] Kiba T, Kudo T, Kojima M, Sakakibara H. Hormonal control of nitrogen acquisition: roles of auxin, abscisic acid, and cytokinin. Journal of experimental botany. 2011;62(4):1399–409. 10.1093/jxb/erq410 21196475

[77] Ristova D, Carré C, Pervent M, Medici A, Kim GJ, Scalia D, et al Combinatorial interaction network of transcriptomic and phenotypic responses to nitrogen and hormones in the Arabidopsis thaliana root. Sci Signal. 2016;9(451):rs13–rs. 10.1126/scisignal.aaf2768 27811143

[78] Ma W, Li J, Qu B, He X, Zhao X, Li B, et al Auxin biosynthetic gene TAR2 is involved in low nitrogen-mediated reprogramming of root architecture in Arabidopsis. The Plant Journal. 2014;78(1):70–9. 10.1111/tpj.12448 24460551

[79] Vanstraelen M, Benková E. Hormonal interactions in the regulation of plant development. Annual review of cell and developmental biology. 2012;28:463–87. 10.1146/annurev-cellbio-101011-155741 22856461

[80] Wang P, Wang Z, Pan Q, Sun X, Chen H, Chen F, et al Growth of maize on mixed nitrate and ammonium promotes auxin synthesis and biomass accumulation. Journal of Experimental Botany. 2019. 10.1093/jxb/erz047 PMC6436159 30759246

[81] Krouk G, Lacombe B, Bielach A, Perrine-Walker F, Malinska K, Mounier E, et al Nitrate-regulated auxin transport by NRT1. 1 defines a mechanism for nutrient sensing in plants. Developmental cell. 2010;18(6):927–37. 10.1016/j.devcel.2010.05.008 20627075

[82] Song W, Sun H, Li J, Gong X, Huang S, Zhu X, et al Auxin distribution is differentially affected by nitrate in roots of two rice cultivars differing in responsiveness to nitrogen. Annals of botany. 2013;112(7):1383–93. 10.1093/aob/mct212 24095838 PMC3806541

[83] Tromas A, Braun N, Muller P, Khodus T, Paponov I, Palme K, et al The AUXIN BINDING PROTEIN 1 is required for differential auxin responses mediating root growth. PloS one. 2009;4:e6648 10.1371/journal.pone.0006648 19777056 PMC2744284

[84] Grones P, Friml J. Auxin transporters and binding proteins at a glance. Journal of Cell Science. 2015;128(1):1–7. 10.1242/jcs.159418 25556248

[85] Bai L, Deng H, Zhang X, Yu X, Li Y. Gibberellin Is Involved in Inhibition of Cucumber Growth and Nitrogen Uptake at Suboptimal Root-Zone Temperatures. PloS one. 2016;11(5):e0156188–e. 10.1371/journal.pone.0156188 27213554 PMC4877016

[86] O'Leary B, Park J, Plaxton WC. The remarkable diversity of plant PEPC (phosphoenolpyruvate carboxylase): recent insights into the physiological functions and post-translational controls of non-photosynthetic PEPCs. Biochemical Journal. 2011;436(1):15–34. 10.1042/BJ20110078 21524275

[87] Zheng Y, Jiao C, Sun H, Rosli HG, Pombo MA, Zhang P, et al iTAK: A Program for Genome-wide Prediction and Classification of Plant Transcription Factors, Transcriptional Regulators, and Protein Kinases. Mol Plant. 2016;9(12):1667–70. 10.1016/j.molp.2016.09.014 27717919

[88] Khan MIR, Trivellini A, Fatma M, Masood A, Francini A, Iqbal N, et al Role of ethylene in responses of plants to nitrogen availability. Front Plant Sci. 2015;6:927–. 10.3389/fpls.2015.00927 26579172 PMC4626634

[89] Vogel MO, Gomez-Perez D, Probst N, Dietz KJ. Combinatorial signal integration by APETALA2/Ethylene Response Factor (ERF)-transcription factors and the involvement of AP2-2 in starvation response. Int J Mol Sci. 2012;13(5):5933–51. 10.3390/ijms13055933 22754341 PMC3382747

[90] Cai H, Xie W, Lian X. Comparative analysis of differentially expressed genes in rice under nitrogen and phosphorus starvation stress conditions. Plant molecular biology reporter. 2013;31(1):160–73.

[91] Takehisa H, Sato Y, Antonio BA, Nagamura Y. Global transcriptome profile of rice root in response to essential macronutrient deficiency. Plant Signal Behav. 2013;8(6):e24409 10.4161/psb.24409 23603969 PMC3907390

[92] Zhao W, Yang X, Yu H, Jiang W, Sun N, Liu X, et al RNA-Seq-based transcriptome profiling of early nitrogen deficiency response in cucumber seedlings provides new insight into the putative nitrogen regulatory network. Plant and Cell Physiology. 2014;56(3):455–67. 10.1093/pcp/pcu172 25432971

[93] Tian Q-Y, Sun P, Zhang W-H. Ethylene is involved in nitrate-dependent root growth and branching in Arabidopsis thaliana. New Phytologist. 2009;184(4):918–31. 10.1111/j.1469-8137.2009.03004.x 19732351

[94] Caba J, Recalde L, Ligero F. Nitrate‐induced ethylene biosynthesis and the control of nodulation in alfalfa. Plant, Cell & Environment. 1998;21(1):87–93.

[95] Nandwal AS, Maan A, Kundu BS, Sheokand S, Kamboj DV, Sheoran A, et al Ethylene evolution and antioxidant defence mechanism in Cicer arietinum roots in the presence of nitrate and aminoethoxyvinylglycine. Plant Physiology and Biochemistry. 2000;38(9):709–15.

