# Correction: Comparative analysis of tissue-specific transcriptomic responses to nitrogen stress in spinach *(Spinacia oleracea)*

**DOI:** 10.1371/journal.pone.0303546

**Published:** 2024-05-07

**Authors:** The PLOS One Staff

In the Plant material and growth conditions subsection of Materials and Methods, there is an error in the first paragraph. The correct paragraph is: The spinach plants were grown in an environmentally controlled growth chamber at the Texas A&M AgriLife Research and Extension Center, Uvalde, Texas. The seeds of spinach variety ‘Banjo’ were grown in plastic containers containing Turface ^®^ based growing medium under 200 μmol m^-2^s^-1^ light intensity (12 hours each light and dark period) at 23°C and 60–70% relative humidity. Two treatments, high N and low N, where N was added in the form of Ca(NO_3_)_2_, were used to maintain the concentration of 200 ppm for high N and 50 ppm for low N. CaCl_2_ was compensated to keep the same concentration of calcium in both treatments. The concentrations of remaining macro- and micro-elements were kept constant using N-free Hoagland’s nutrient solution (No. 2 Basal Salt Mixture, Caisson Labs, Smithfield, UT, USA) comprising 2.86 mg L^−1^ H_3_BO_3_, 554.90 mg L^−1^ CaCl_2_, 0.045 mg L^−1^ CuCl_2_, 33.00 mg L^−1^ C_10_H_12_N_2_NaFeO_8_ 3H_2_O, 240.33 mg L^−1^ MgSO_4_, 1.81 mg L^−1^ MnCl_2_ 4H_2_O, 0.025 mg L^−1^ Na_2_MoO_4_ 2H_2_O, 372.70 mg L^−1^ KCl, 136.025 mg L^−1^ KH_2_PO_4_, and 0.1 mg L^−1^ ZnCl_2_. Samples collected from 6-week-old plants were frozen in liquid nitrogen and stored at −80°C until subsequent analyses. Three independent plants were used for metabolic analysis, total RNA extractions, physiological and biochemical analysis.

In the Determination of physiological traits, N, and free amino acids subsection of Materials and Methods, there is an error in the fourth sentence. The correct sentence is: The amino acid analysis was performed using Waters ^™^ Acquity^™^ UPLC^™^ H-class system coupled to Waters Xevo Xevo TQ-S MS-MS mass spectrometer with electrospray ionization (ESI) probe following pre-established protocol [35].

The publisher apologizes for the errors.
