# Supplementary figures and images for: Comparative analysis of tissue-specific transcriptomic responses to nitrogen stress in spinach (Spinacia oleracea)
Source: PLoS One. 2020 May 6;15(5):e0232011. doi: 10.1371/journal.pone.0232011 (PMC7202632; doi:10.1371/journal.pone.0232011)

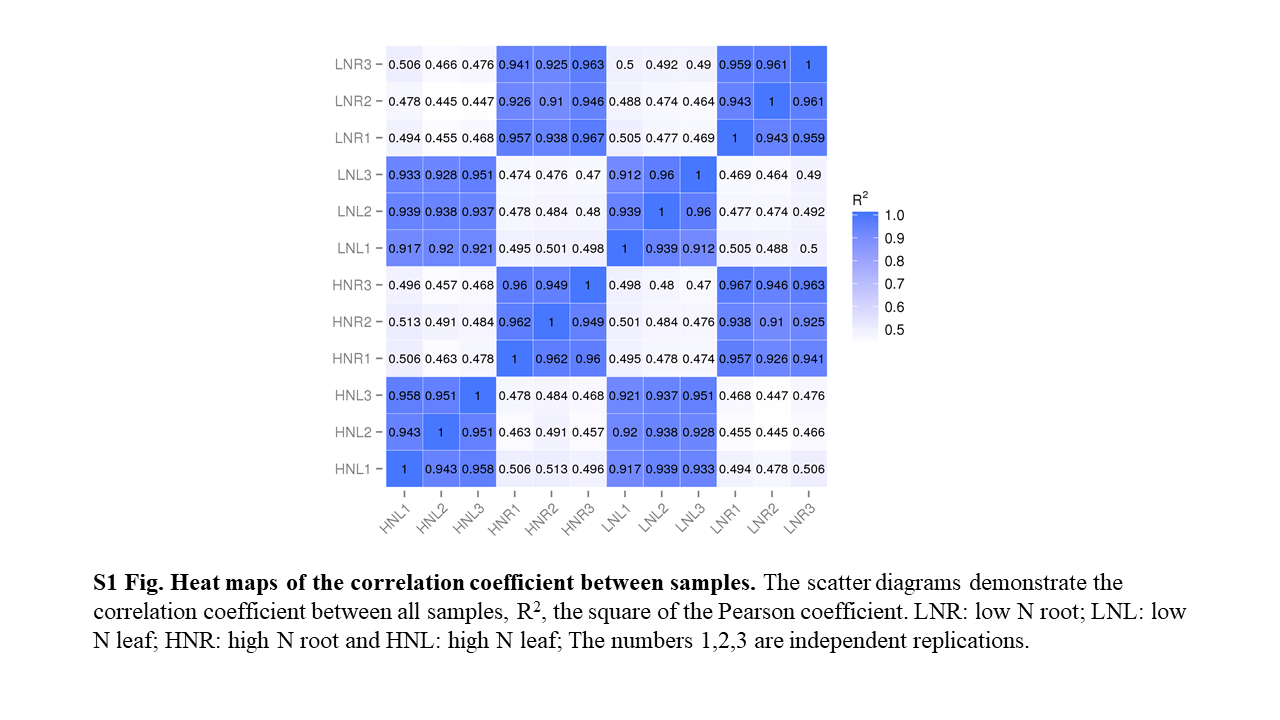

Supplement: S1 Fig — The scatter diagrams demonstrate the correlation coefficient between all samples, R2, the square of the Pearson coefficient. LNR: low N root; LNL: low N leaf; HNR: high N root and HNL: high N leaf; The numbers 1,2,3 are independent replications. (TIF) [file pone.0232011.s001.TIF]

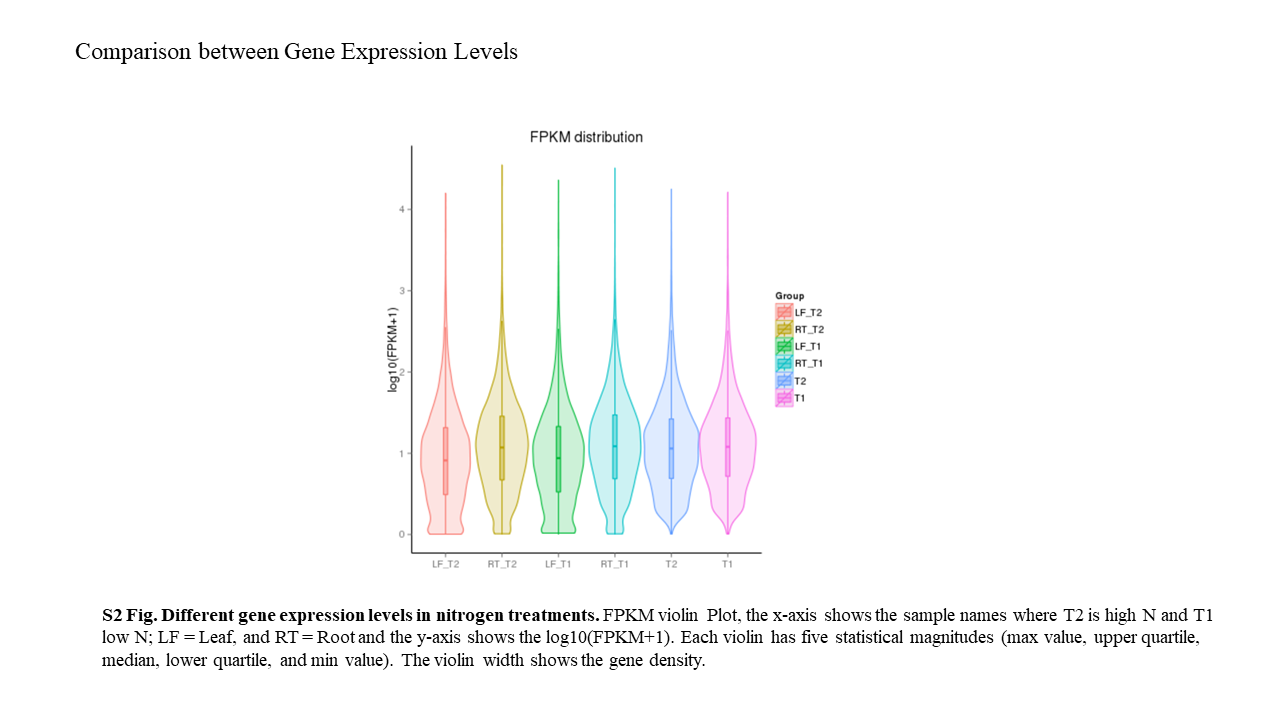

Supplement: S2 Fig — FPKM violin Plot, the x-axis shows the sample names where T2 is high N and T1 low N; LF = Leaf and RT = Root, and the y-axis shows the log10(FPKM+1). Each violin has five statistical magnitudes (max value, upper quartile, median, lower quartile, and min value). The violin width shows the gene density. (TIF) [file pone.0232011.s002.TIF]

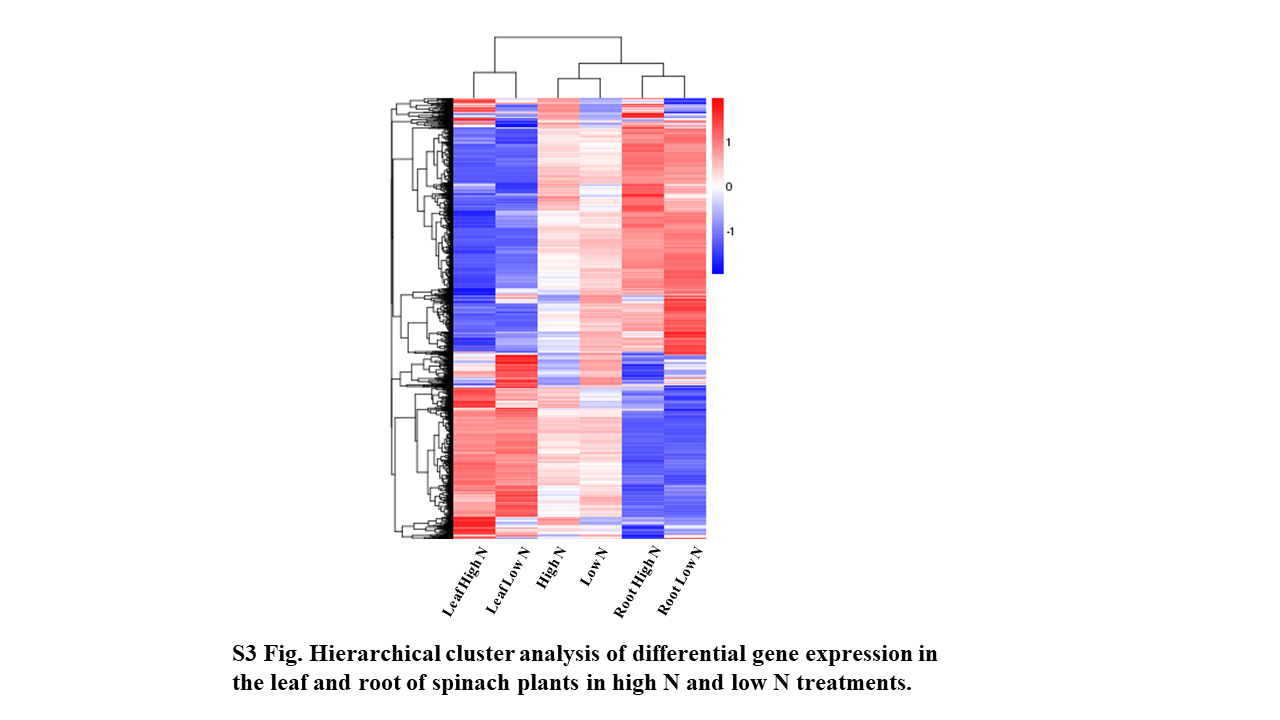

Supplement: S3 Fig — (TIF) [file pone.0232011.s003.TIF]

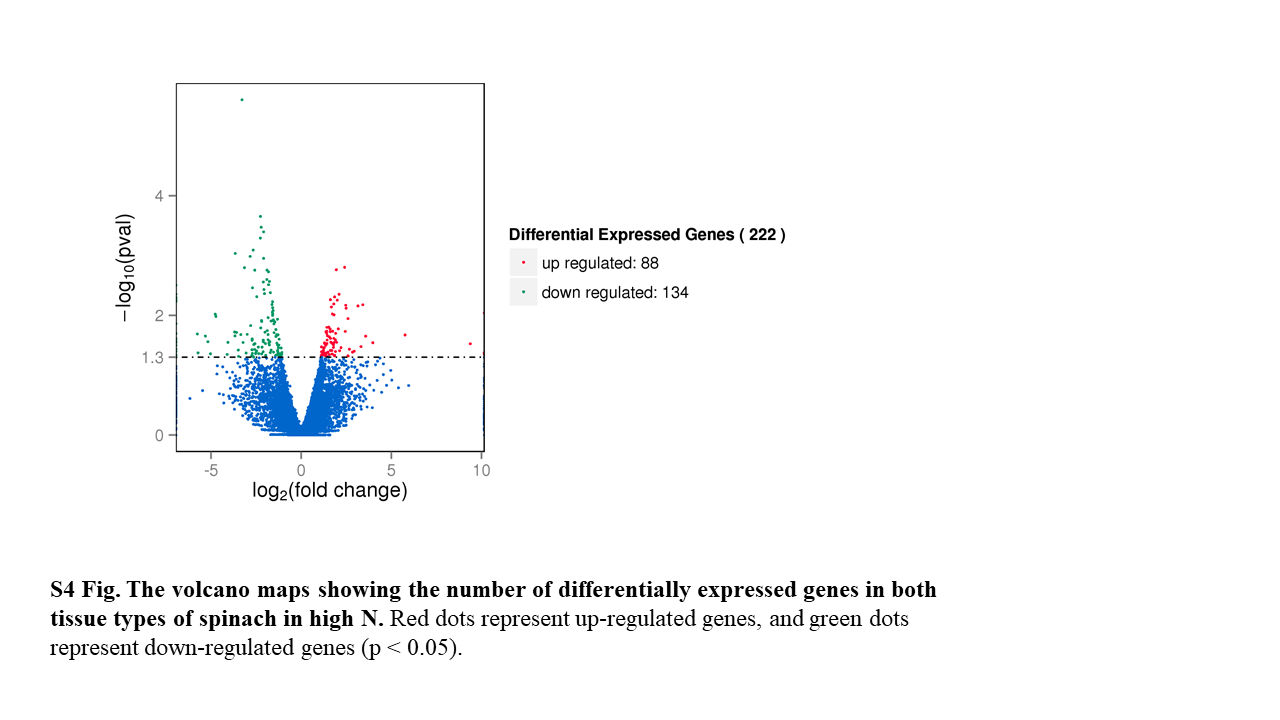

Supplement: S4 Fig — Red dots represent up-regulated genes, and green dots represent down-regulated genes (p < 0.05). (TIF) [file pone.0232011.s004.TIF]

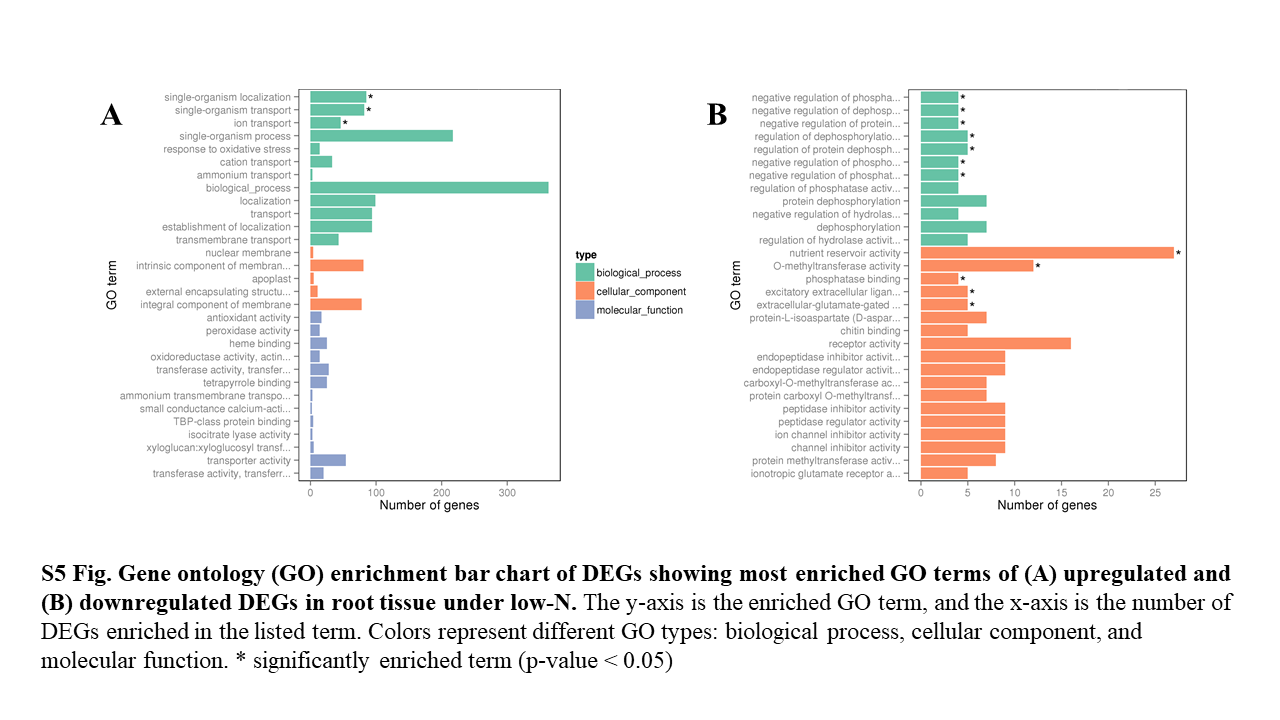

Supplement: S5 Fig — Gene ontology (GO) enrichment bar chart of DEGs showing most enriched GO terms of (A) upregulated and (B) downregulated DEGs in root tissue under Low-N. The y-axis is the enriched GO term, and the x-axis is the number of DEGs enriched in the listed term. Colors represent different GO types: biological process, cellular component, and molecular function. * significantly enriched term (p-value < 0.05). (TIF) [file pone.0232011.s005.TIF]

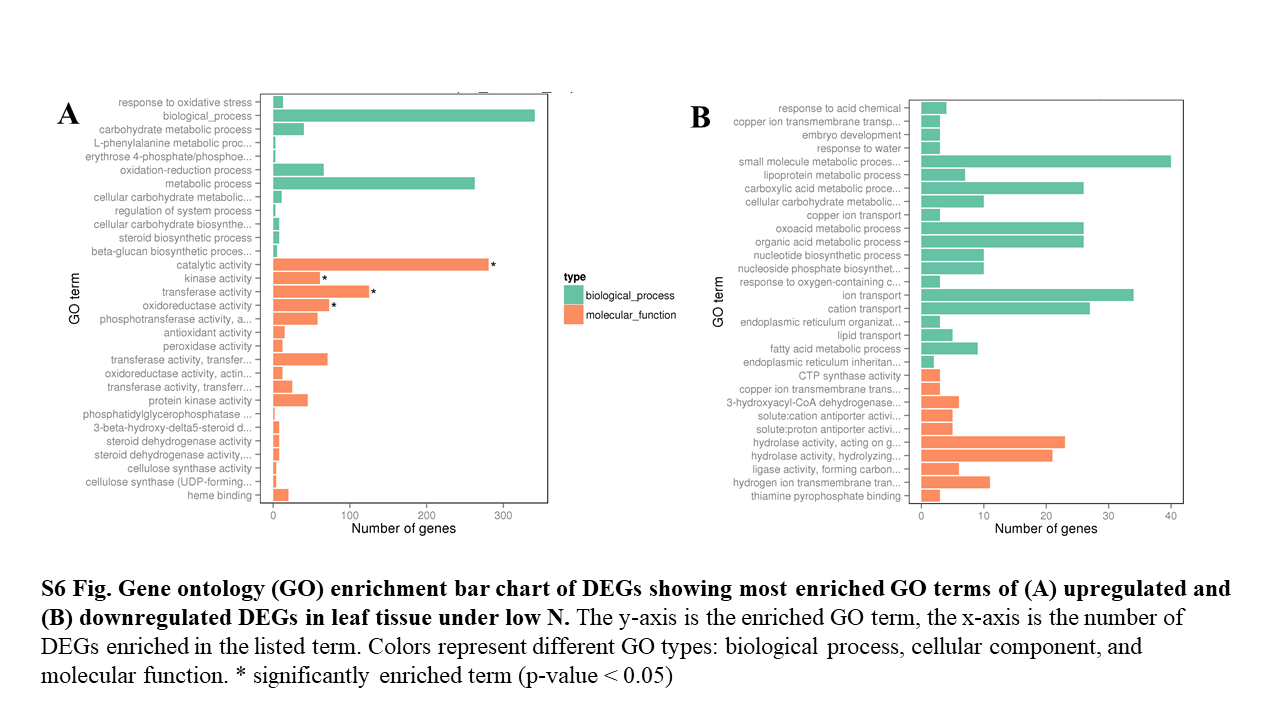

Supplement: S6 Fig — Gene ontology (GO) enrichment bar chart of DEGs showing most enriched GO terms of (A) upregulated and (B) downregulated DEGs in leaf tissue under low N. The y-axis is the enriched GO term, the x-axis is the number of DEGs enriched in the listed term. Colors represent different GO types: biological process, cellular component, and molecular function. * significantly enriched term (p-value < 0.05). (TIF) [file pone.0232011.s006.TIF]

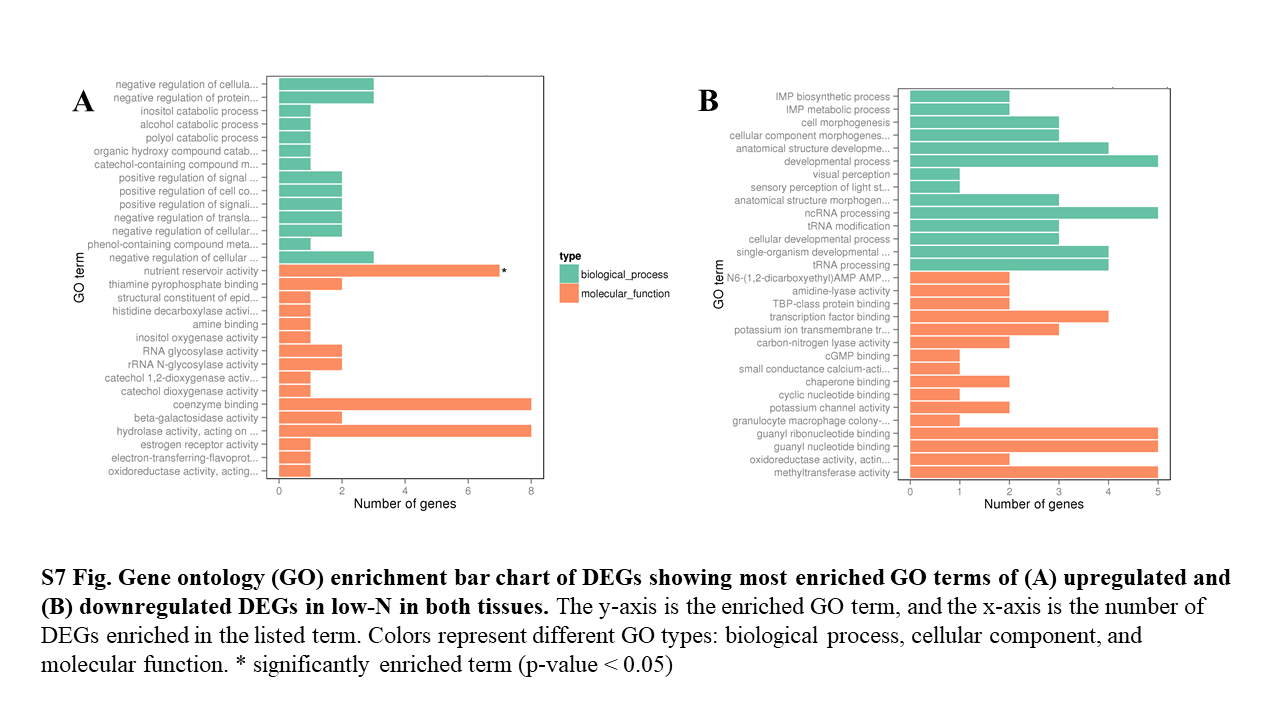

Supplement: S7 Fig — The y-axis is the enriched GO term, and the x-axis is the number of DEGs enriched in the listed term. Colors represent different GO types: biological process, cellular component, and molecular function. * significantly enriched term (p-value < 0.05) (TIF) [file pone.0232011.s007.TIF]

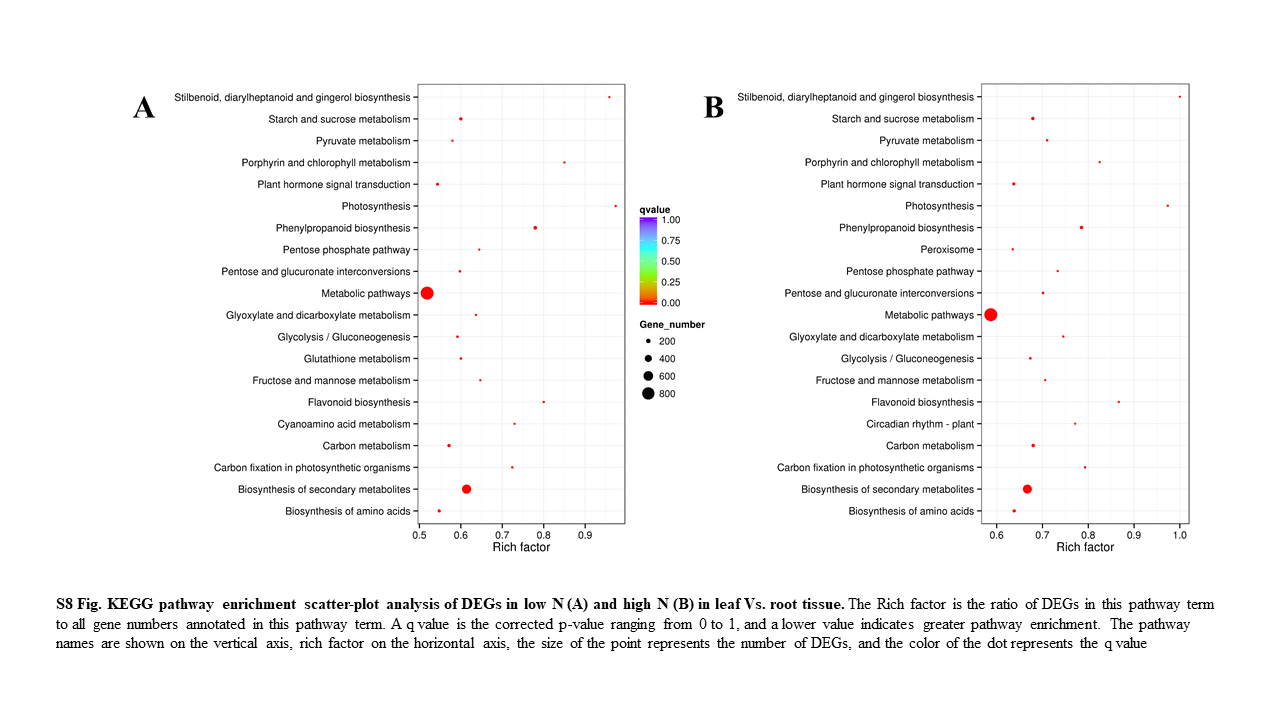

Supplement: S8 Fig — KEGG pathway enrichment scatter-plot analysis of DEGs in low N (A) and high N (B) in leaf Vs. root tissue. The Rich factor is the ratio of DEGs in this pathway term to all gene numbers annotated in this pathway term. A q value is the corrected p-value ranging from 0 to 1, and a lower value indicates greater pathway enrichment. The pathway names are shown on the vertical axis, rich factor on the horizontal axis, the size of the point represents the number of DEGs, and the color of the dot represents the q value (TIF) [file pone.0232011.s008.TIF]

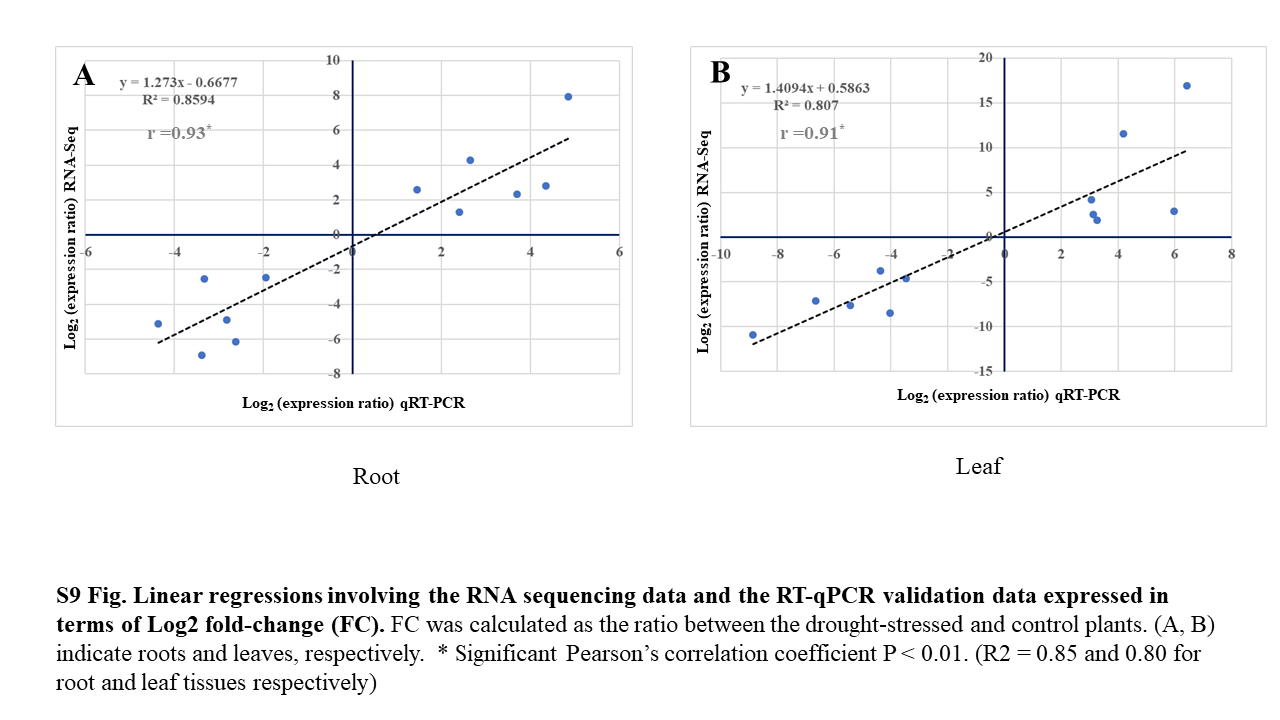

Supplement: S9 Fig — FC was calculated as the ratio between the drought-stressed and control plants. (A, B) indicate roots and leaves, respectively. * Significant Pearson’s correlation coefficient P < 0.01. (R2 = 0.85 and 0.80 for root and leaf tissues respectively). (TIF) [file pone.0232011.s009.TIF]

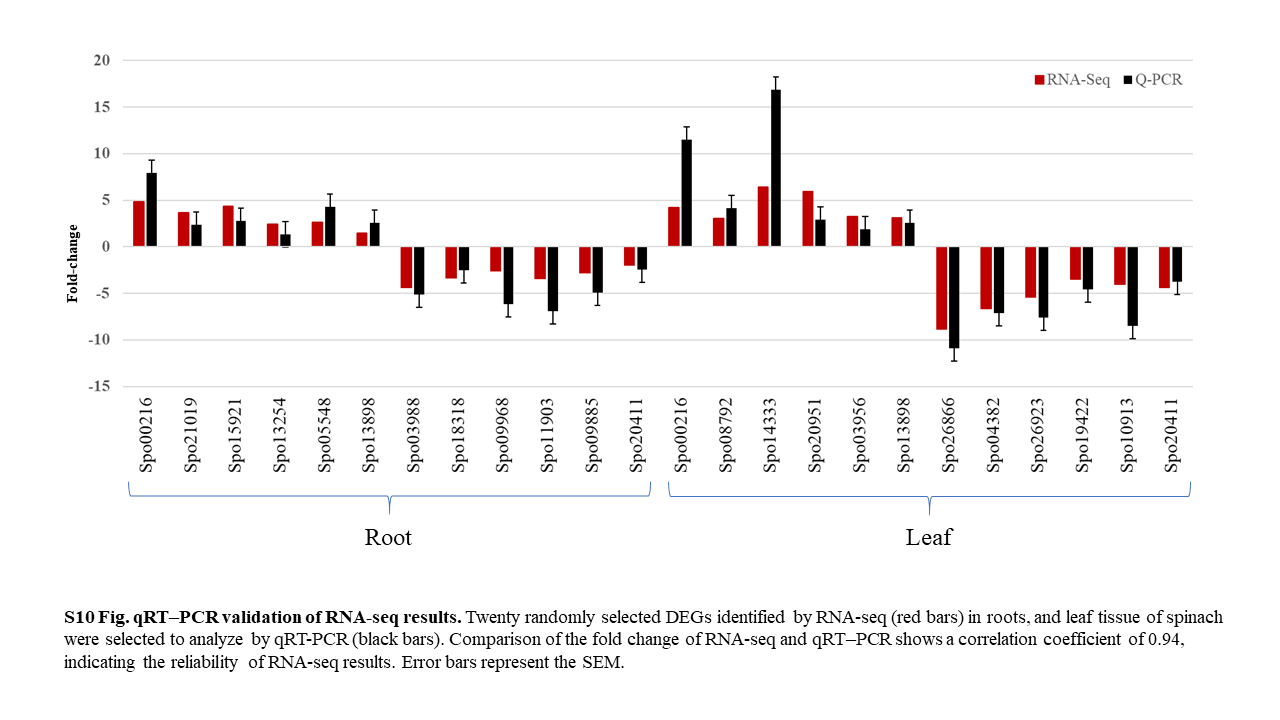

Supplement: S10 Fig — Twenty randomly selected DEGs identified by RNA-seq (red bars) in roots, and leaf tissue of spinach were selected to analyze by qRT-PCR (black bars). Comparison of the fold change of RNA-seq and qRT–PCR shows a correlation coefficient of 0.94, indicating the reliability of RNA-seq results. Error bars represent the SEM. (TIF) [file pone.0232011.s010.TIF]

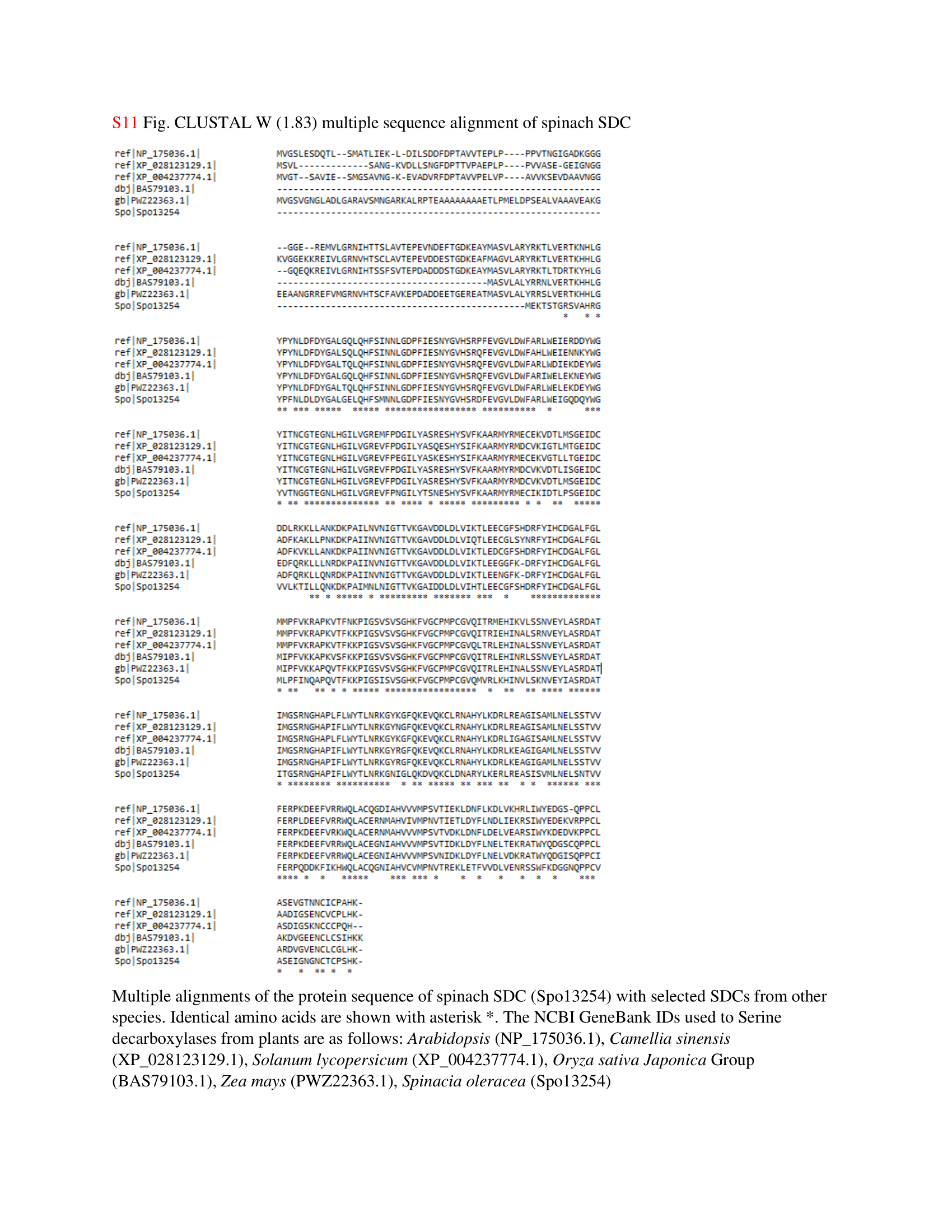

Supplement: S11 Fig — Multiple alignments of the protein sequence of spinach SDC (Spo13254) with selected SDCs from other species. Identical amino acids are shown with asterisk *. The NCBI GeneBank IDs used to Serine decarboxylases from plants are as follows: Arabidopsis (NP_175036.1), Camellia sinensis (XP_028123129.1), Solanum lycopersicum (XP_004237774.1), Oryza sativa Japonica Group (BAS79103.1), Zea mays (PWZ22363.1), Spinacia oleracea (Spo13254) (TIFF) [file pone.0232011.s011.tiff]

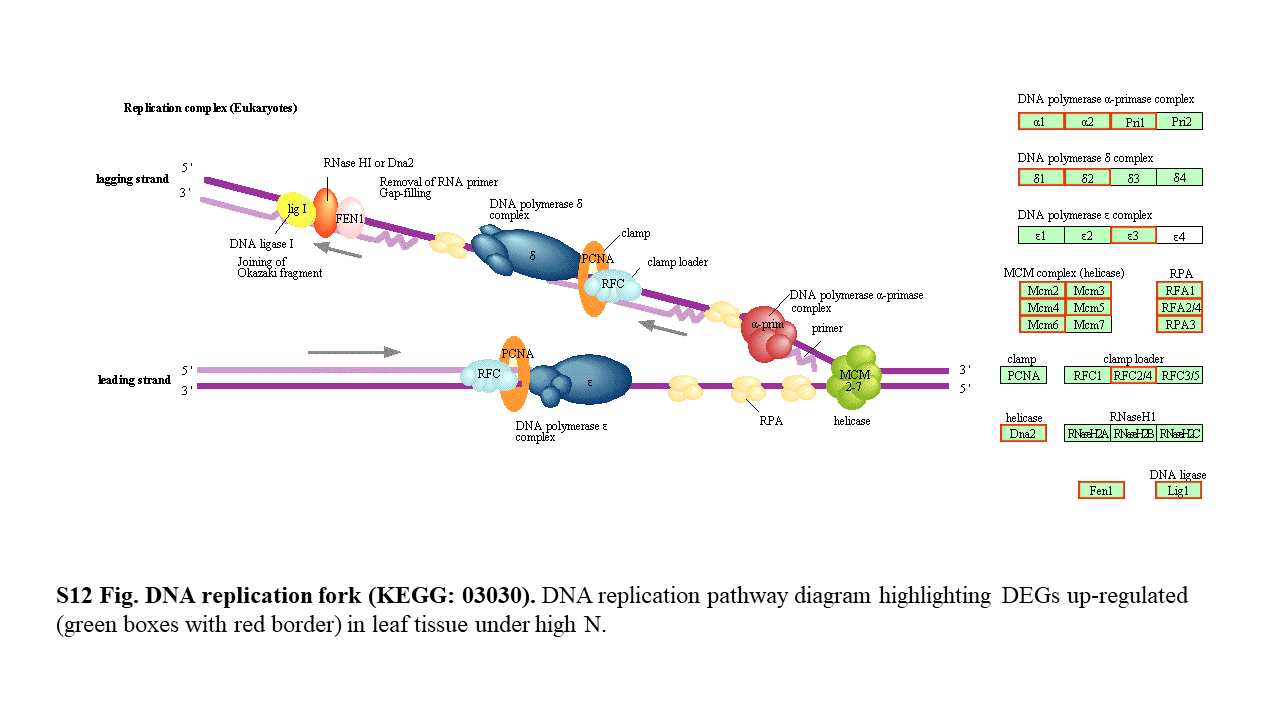

Supplement: S12 Fig — DNA replication pathway diagram highlighting DEGs up-regulated (green boxes with red border) in leaf tissue under high N. (TIF) [file pone.0232011.s012.TIF]

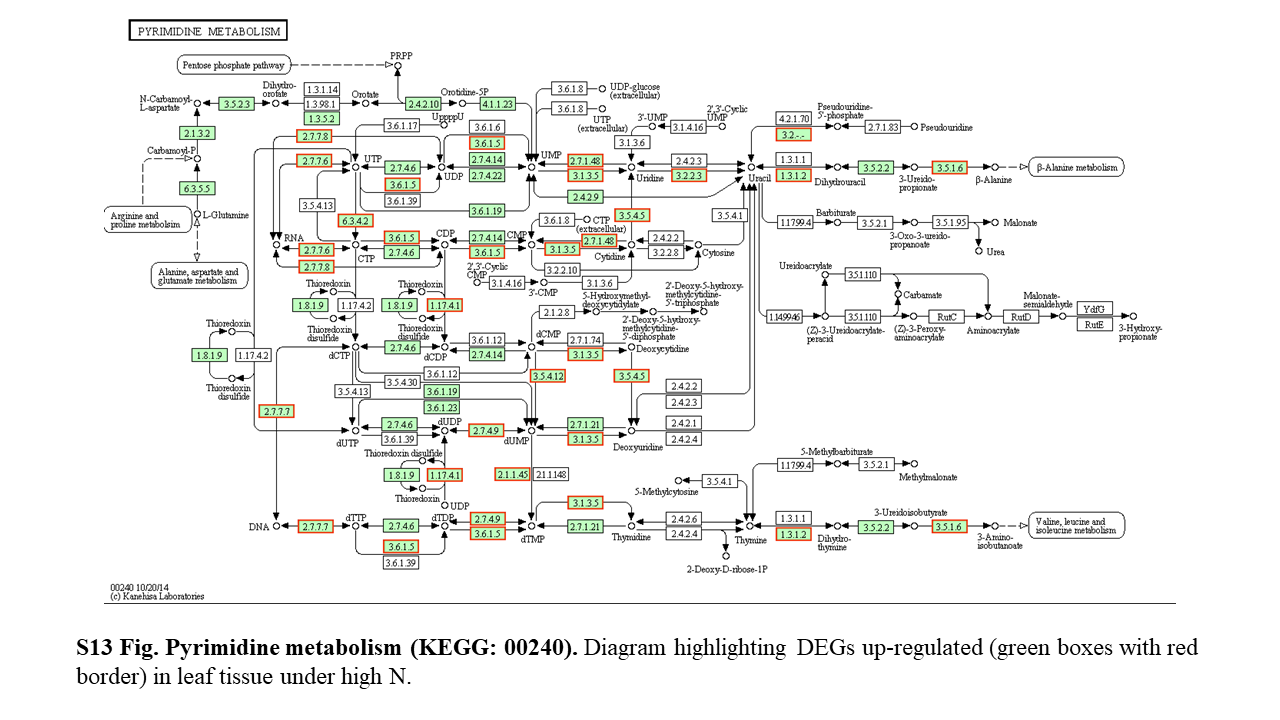

Supplement: S13 Fig — Diagram highlighting DEGs up-regulated (green boxes with red border) in leaf tissue under high N. (TIF) [file pone.0232011.s013.TIF]

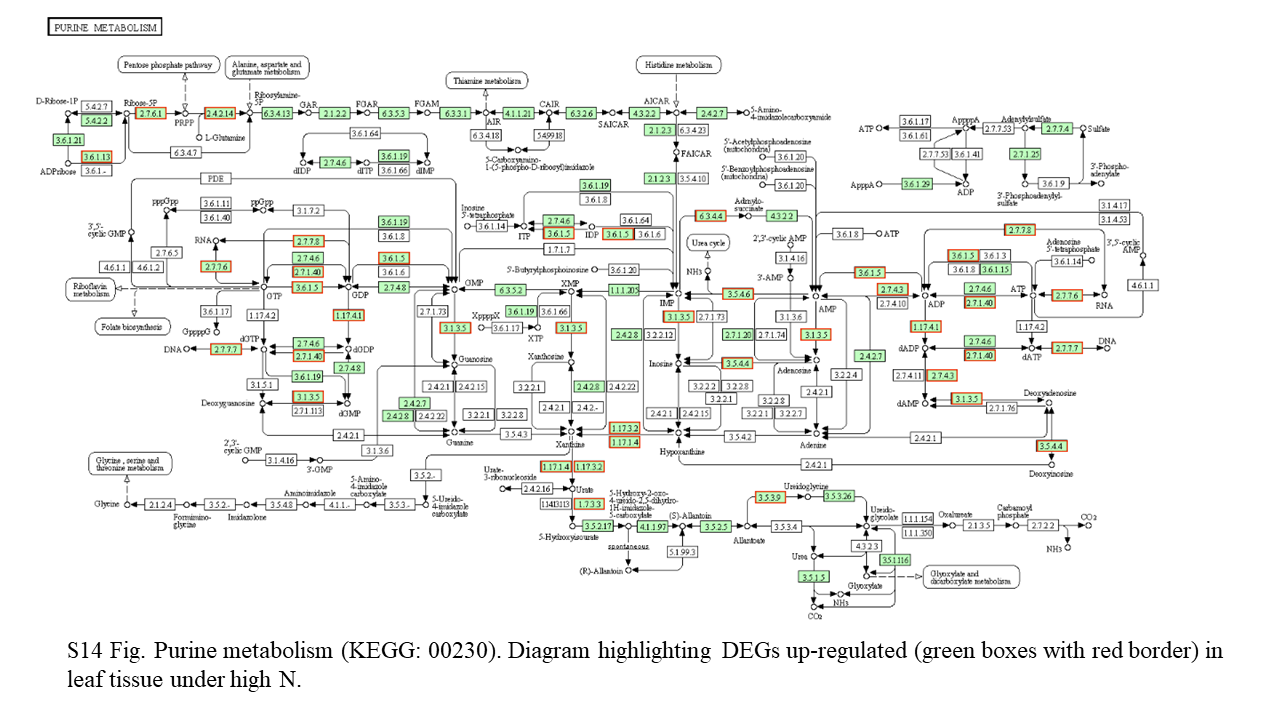

Supplement: S14 Fig — Diagram highlighting DEGs up-regulated (green boxes with red border) in leaf tissue under high N. (TIF) [file pone.0232011.s014.TIF]
